# Regulation of *N*-Formyl Peptide Receptor Signaling and Trafficking by Arrestin-Src Kinase Interaction

**DOI:** 10.1371/journal.pone.0147442

**Published:** 2016-01-20

**Authors:** Brant M. Wagener, Nicole A. Marjon, Eric R. Prossnitz

**Affiliations:** Department of Internal Medicine and UNM Comprehensive Cancer Center, University of New Mexico Health Sciences Center, Albuquerque, NM, United States of America; Loyola University Chicago, Stritch School of Medicine, UNITED STATES

## Abstract

Arrestins were originally described as proteins recruited to ligand-activated, phosphorylated G protein-coupled receptors (GPCRs) to attenuate G protein-mediated signaling. It was later revealed that arrestins also mediate GPCR internalization and recruit a number of signaling proteins including, but not limited to, Src family kinases, ERK1/2, and JNK3. GPCR-arrestin binding and trafficking control the spatial and temporal activity of these multi-protein complexes. In previous reports, we concluded that *N*-formyl peptide receptor (FPR)-mediated apoptosis, which occurs upon receptor stimulation in the absence of arrestins, is associated with FPR accumulation in perinuclear recycling endosomes. Under these conditions, inhibition of Src kinase and ERK1/2 prevented FPR-mediated apoptosis. To better understand the role of Src kinase in this process, in the current study we employed a previously described arrestin-2 (arr2) mutant deficient in Src kinase binding (arr2-P91G/P121E). Unlike wild type arrestin, arr2-P91G/P121E did not inhibit FPR-mediated apoptosis, suggesting that Src binding to arrestin-2 prevents apoptotic signaling. However, in cells expressing this mutant, FPR-mediated apoptosis was still blocked by inhibition of Src kinase activity, suggesting that activation of Src independent of arrestin-2 binding is involved in FPR-mediated apoptosis. Finally, while Src kinase inhibition prevented FPR-mediated-apoptosis in the presence of arr2-P91G/P121E, it did not prevent FPR-arr2-P91G/P121E accumulation in the perinuclear recycling endosome. On the contrary, inhibition of Src kinase activity mediated the accumulation of activated FPR-wild type arrestin-2 in recycling endosomes without initiating FPR-mediated apoptosis. Based on these observations, we conclude that Src kinase has two independent roles following FPR activation that regulate both FPR-arrestin-2 signaling and trafficking.

## Introduction

Arrestins were originally described as cytosolic proteins recruited to ligand-activated and phosphorylated G protein-coupled receptors (GPCRs) [1–3]. Through direct binding interactions with phosphorylated GPCRs, arrestins sterically block the binding and activation of heterotrimeric G proteins, thus inhibiting the GDP-GTP exchange of Gα subunits and abating G protein-initiated signaling cascades, resulting in desensitization [4]. Subsequently, arrestins were demonstrated to mediate GPCR internalization [5,6] with binding sites for adaptor protein (AP)-2 and clathrin identified within the C-terminus of arrestins [7–10]. Classical GPCRs such as the beta2-adreneric receptor (β2-AR) [11] and the angiotensin II (Type 1A) receptor (AT1_A_R) [12] require arrestin and the subsequent binding of AP-2 and clathrin for internalization [13,14]. However, multiple GPCRs, including but not limited to the *N*-formyl peptide receptor (FPR) [15] and the m2-muscarinic acetylcholine receptor [16], do not require arrestins for ligand-dependent internalization.

More recently, arrestins have been described as scaffolds for G protein-independent GPCR-mediated signaling complexes [17–19]. Signaling proteins, including Src kinase [19,20], ERK1/2 [19,21] and JNK3 [22–24], bind arrestins and are activated by ligand- and arrestin-bound GPCRs. It has been proposed that arrestins selectively recruit signaling mediators to activated and internalized GPCR signaling scaffolds to prevent crosstalk between unintended proteins through the spatial and temporal localization of signaling complexes [25]. For example, following β2-AR activation in the absence of arrestin binding, ERK1/2 is phosphorylated and translocates to the nucleus where it activates downstream effectors leading to alterations in gene expression [21]. However, when ERK1/2 is phosphorylated in the context of a GPCR-arrestin scaffold, activated ERK1/2 remains localized to endosome-localized receptors and activates cytosolic effector molecules [19,26,27].

Of the aforementioned signaling scaffolds, arrestin-Src kinase interactions are among the better described, with important functions in cell migration and degranulation [19,28]. In addition, disruption of the interaction between arrestin-2 and Src kinase, by mutation of arrestins, decreases the phosphorylation of ERK1/2 in response to β2-AR and 5-HT_1A_R but not AT1_A_R stimulation [20]. Finally, an interaction between arrestin-2 and Src kinase is required to phosphorylate tyrosine residues in β2-adaptin, facilitating the release of AP-2 from arrestin, a process required for efficient AT1_A_R internalization and trafficking [12,29].

In the absence of arrestins, as in murine embryonic fibroblasts (MEFs) derived from arrestin-2/3 double knockout mice, although most GPCRs no longer internalize [30], activation of the chemoattractant *N*-formyl peptide receptor (FPR, [31,32]) results in unaltered receptor internalization but defective recycling, resulting in accumulation in the Rab11-containing perinuclear endosomal compartment [15], followed by the rapid initiation of apoptosis [33]. FPR-mediated apoptosis is prevented by reconstitution of cells with either wild type arrestin-2 or -3, or pretreating cells with inhibitors of Src family kinases, ERK1/2, JNK3 or p38 [33]. Finally, arrestin-2 mutants that exhibit defective binding to AP-2 did not rescue apoptosis in arrestin-deficient cells and similarly did not rescue the recycling deficit, resulting in the continued accumulation of the FPR in perinuclear endosomes and the induction of apoptosis [34].

Whereas arrestins control early signaling and trafficking events for many model GPCRs [4,6,19], it has become apparent that they also regulate post-endocytic signaling and trafficking of the FPR [34]. As the interaction between arrestin-2 and Src kinase can mediate GPCR internalization via AP-2 and activation of ERK1/2, we hypothesized that reduced binding of Src kinase to arrestin-2 could lead to FPR accumulation in perinuclear recycling endosomes and the aberrant activation of ERK1/2, thereby initiating apoptosis as in arrestin-deficient cells. To this end, we employed a mutant of arrestin-2 (arr2-P91G/P121E) that exhibits decreased binding to Src kinase in response to β2-AR activation [19,20] to better understand the role of the arrestin-2-Src kinase interaction in FPR trafficking and signaling. Our results demonstrate two independent roles for Src kinase in its association with arrestin-2 that independently regulate both signaling and trafficking of the FPR.

## Results

### An arrestin mutant deficient in Src kinase binding does not reverse FPR-mediated apoptosis

We have previously demonstrated that in the absence of arrestin, FPR-mediated apoptosis is prevented by inhibitors of p38, ERK1/2, JNK or Src family kinases [33]. This is consistent with the concept that arrestins serve as protein scaffolds that control temporal and spatial aspects of signaling following GPCR activation in part through the selective recruitment of such downstream kinases [35]. To better understand how arrestins regulate signaling within the context of FPR activation and apoptotic signaling, we employed a mutant of arrestin-2 that is deficient in Src kinase binding (arr2-P91G/P121E) [20]. This mutant does not support ERK1/2 phosphorylation in response to β2-AR activation and inhibits internalization of the β2-AR [19,20].

**Fig 1:**
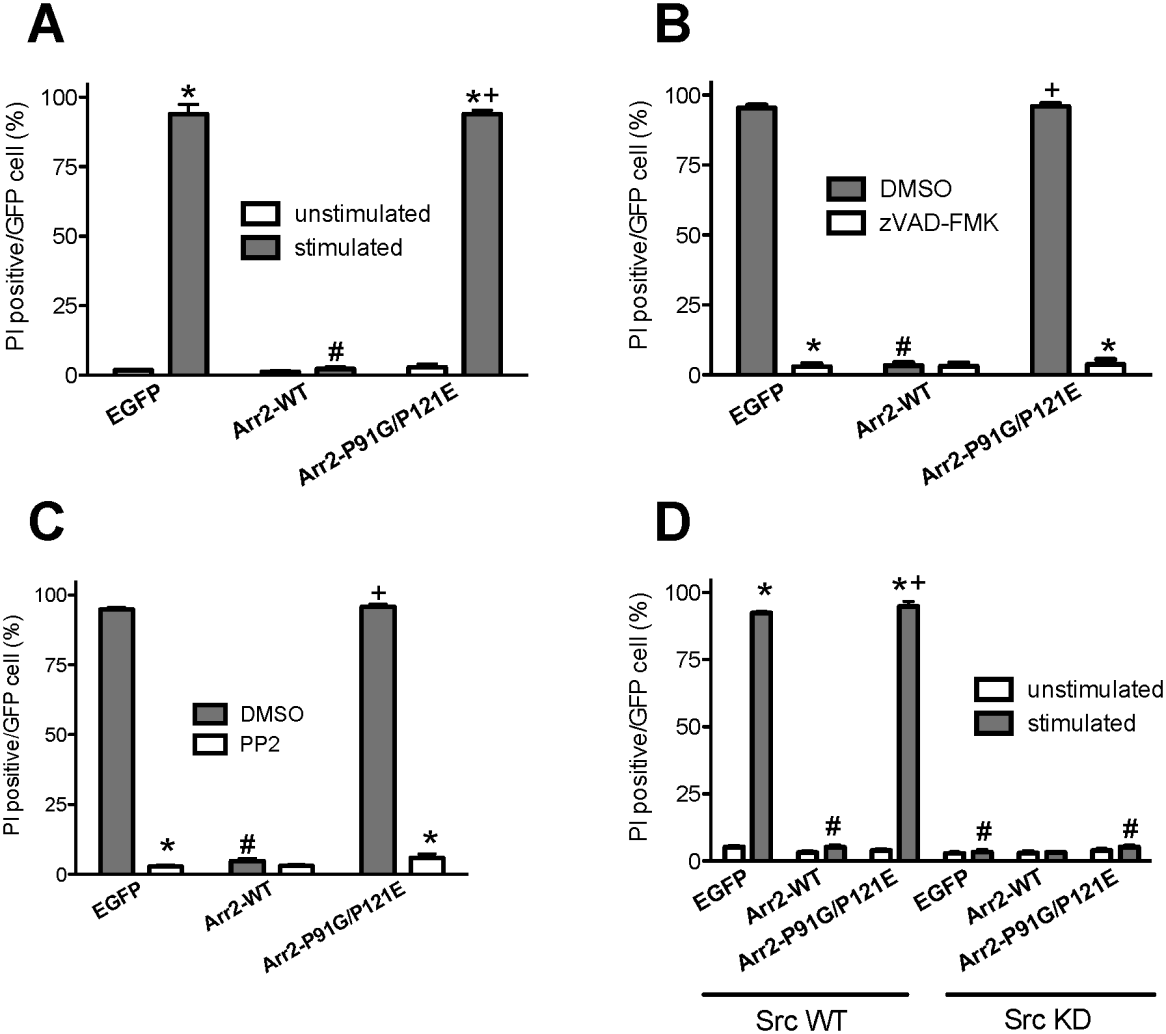
Inhibition of Src kinase, but not expression of Arr2-P91G/P121E, prevents FPR-mediated apoptosis. Arr-2^-/-^/-3^-/-^ FPR cells were transiently transfected with the indicated constructs, stimulated with 10 nM fMLF and stained with PI. Random fields were viewed by fluorescence microscopy until 100–300 GFP-expressing cells were evaluated for PI staining. **A)** Arr-2^-/-^/-3^-/-^ FPR cells were transiently transfected with the indicated GFP-fused arrestins or vector only (EGFP) and treated with 10 nM fMLF (stimulated) or vehicle (unstimulated). **B)** Arr-2^-/-^/-3^-/-^ FPR cells were transiently transfected with the indicated GFP plasmids as in **A** and incubated with DMSO or zVAD-FMK (10 nM, 30 min) before and during stimulation with 10 nM fMLF. **C)** Arr-2^-/-^/-3^-/-^ FPR cells were transiently transfected with the indicated plasmids and incubated with DMSO or PP2 (10 nM, 30 min) before and during stimulation with 10 nM fMLF. **D)** Arr-2^-/-^/-3^-/-^ FPR cells were transiently co-transfected with the indicated arrestin plasmids and either wild type Src kinase or kinase dead (K298M) Src kinase, followed by treatment with 10 nM fMLF (stimulated) or vehicle (unstimulated). Data are expressed as the mean percentage of PI positive/GFP cell +/- SEM from three independent experiments. * p<0.001 vs. unstimulated (A, D) or DMSO-treated (B, C) cells; # p<0.001 vs. EGFP-transfected cells (A-C), EGFP-transfected Src WT cells (D) or arr2-P91G/P121E-transfected cells (D) as appropriate; + p<0.001 vs. arr2-WT-transfected cells.

To determine whether the arrestin-2/Src kinase interaction plays a role in FPR signaling, we assessed the arr2-P91G/P121E mutant for its ability to regulate FPR-mediated apoptosis. We utilized mouse embryonic fibroblasts deficient in arrestin-2 and -3 (derived from arrestin-2^-/-^-3^-/-^ double knockout mice) [30] stably expressing the FPR (designated as arr-2^-/-^-3^-/-^ FPR MEF) to assess the consequences of FPR activation in the presence or absence of transiently transfected wild type or mutant arrestins (as GFP fusion proteins) [33,34]. The advantage of this system is the complete absence of endogenous wild type arrestins that could mitigate the effects of the transfected arrestin constructs. In the absence of ligand activation, less than 5% of cells expressing any of the GFP vectors (EGFP vector only, arr2-WT or arr2-P91G/P121E) underwent apoptosis as determined by propidium iodide (PI) staining (Fig 1A), consistent with our previous work demonstrating that apoptosis in these cells is dependent upon FPR activation [33,34]. On the contrary, greater than 90% of arr-2^-/-^-3^-/-^ FPR MEFs expressing the EGFP vector (no arrestin present) stained with PI when stimulated with the agonist fMLP. Furthermore, when the FPR was stimulated in the presence of transfected wild-type arrestin-2-GFP, less than 5% of cells were again stained with PI. Finally, FPR activation in the presence of arr2-P91G/P121E-GFP resulted in extensive staining with PI, similar to the vector-only transfected cells. These results suggest that an interaction between arrestin-2 and Src kinase is necessary to ensure appropriate FPR signaling with respect to apoptosis.

Our previous reports have described multiple aspects of FPR-mediated apoptosis, including caspase activation [33,34]. To confirm that the observed PI staining in the presence of arr2-P91G/P121E was apoptosis resulting from caspase activation, arr-2^-/-^/-3^-/-^ FPR MEFs expressing GFP-fused arrestins were assayed for PI staining in the presence of the pan-caspase inhibitor zVAD-FMK (Fig 1B). In the presence of the caspase inhibitor, less than 5% of the EGFP- and arr2-P91G/P121E-GFP- expressing cells were stained with PI, while greater than 90% of cells stained in the presence of vehicle only. These data establish that activation of the FPR, when not regulated by wild type arrestin-2, leads to the initiation of caspase-dependent apoptosis [33,34].

### Inhibition of Src kinase activity blocks FPR-mediated apoptosis in the absence of Src binding to arrestin

Although previous publications have reported that overexpression of arr2-P91G/P121E inhibited ERK1/2 activation in response to β2-AR activation, this mutant did not affect ERK1/2 activation by the AT1_A_R [20]. These results suggest that that the requirement for Src association with arrestin in ERK1/2 signaling is dependent on the receptor system involved. We therefore asked whether expression of the Src binding-deficient P91G/P121E arrestin mutant would prevent Src kinase activation or instead induce aberrant activation of Src kinase leading to FPR-mediated apoptosis. To determine if Src kinase activity was required to initiate apoptosis in response to FPR stimulation, independent of arrestin-2 binding, we used the Src family kinase inhibitor, PP2 (Fig 1C). In arr-2^-/-^/-3^-/-^ FPR MEFs expressing EGFP and arr2-P91G/P121E-GFP, less than 5% of transfected FPR-stimulated cells stained with PI in the presence of PP2, compared to greater than 90% in the presence of vehicle only. In the arr-2^-/-^/-3^-/-^ FPR MEFs expressing arr2-WT-GFP, PP2 had no effect on the extent of FPR-initiated apoptosis. These data indicate that even in the absence of Src binding to arrestin (expression of arr2-P91G/P121E), Src kinase activity is still required for FPR-mediated apoptosis.

As PP2 is an inhibitor of multiple Src family kinases [36], we further investigated whether our results were specifically due to inhibition of Src kinase activity or potentially one of the other family members acted upon by this inhibitor. Accordingly, arr-2^-/-^/-3^-/-^ FPR MEFs were cotransfected with GFP-fused arrestins and either wild type or kinase dead (K298M) Src kinase (Fig 1D). In the presence of wild type Src kinase, only arr-2^-/-^/-3^-/-^ FPR MEFs expressing EGFP or arr2-P91G/P121E stained extensively with PI, while cells expressing arr2-WT did not, consistent with the above results (Fig 1A). In arr-2^-/-^/-3^-/-^ FPR MEFs expressing GFP-fused arrestins (WT, P91G/P121E, or no arrestin/vector only) and kinase dead Src kinase, less than 5% of cells stained with PI in response to FPR activation regardless of the absence or presence of arrestin (wild type or P91G/P121E), suggesting that Src kinase activity was specifically required to induce the apoptotic phenotype (Fig 1D). Although expression of an arrestin mutant with decreased binding to Src kinase does not prevent apoptosis, inhibition of Src kinase activity with PP2 or a kinase dead mutant of Src continued to prevent apoptosis, indicating that Src kinase activity continues to play a role in FPR-mediated apoptosis even in the absence of Src binding to arrestin.

### Inhibition of Src binding to arrestin or Src kinase activity does not alter FPR internalization

We have previously demonstrated that FPR activation in the absence of internalization is insufficient to induce apoptosis in arrestin-deficient cells [33]. Based on these results, we expected that in the presence of the arr2-P91G/P121E Src-binding-deficient mutant, the FPR would continue to internalize upon activation. We therefore determined the level of FPR expression as well as the rate and maximal extent of FPR internalization in the absence and presence of either arr2-WT or arr2-P91G/P121E. Basal FPR cell surface expression levels were not affected by expression of either arr2-WT or arr2-P91G/P121E in arr-2^-/-^/-3^-/-^ FPR MEFs (Table 1). Furthermore, the rate (t_1/2_) of FPR internalization as well as the maximum extent of internalization of the FPR in the presence of the arr2-P91G/P121E mutant were similar to FPR internalization in the presence of EGFP alone or wild type arrestin-2 (Table 1). However, as inhibition of Src kinase activity prevented FPR-mediated apoptosis in the absence of arrestins or the presence of arr2-P91G/P121E-GFP, this effect could be a result of decreased internalization in the presence of Src inhibition. Therefore, we assayed FPR internalization in the presence of GFP-fused arrestins and PP2. Although Src kinase activity was required for FPR-mediated apoptosis in the arr-2^-/-^/-3^-/-^ FPR MEFs [33] or in the presence of arr2-P91G/P121E-GFP (Fig 1C and 1D), PP2 had no effect on the level of FPR expression or the rate or extent of FPR internalization (Table 1). These data demonstrate that altering Src kinase signaling does not prevent apoptosis through inhibition of FPR internalization, and that FPR internalization itself occurs independently of Src kinase binding or activity.

**Table 1:** FPR expression and internalization in the absence and presence of arrestins.

Transfected Vector and Treatment	Basal FPR expression (%)^a^	Internalization t _1/2_ (min)	Maximum Internalization (%)
EGFP only	100	8.9 ± 1.3	72 ± 13.5
Arr2-WT	96 ± 3	5.7 ± 2.4	83 ± 15.5
Arr2-P91G/P121E	99 ± 4	4.8 ± 1.7	69 ± 12.5
EGFP+DMSO	102 ± 2	6.3 ± 1.5	67 ± 13.7
Arr2-WT+DMSO	101 ± 5	4.6 ± 1.0	62 ± 15.2
Arr2-P91G/P121E+DMSO	104 ± 2	5.4 ± 1.2	78 ± 7.5
EGFP+PP2	97 ± 3	6.0 ± 1.7	63 ± 10.7
Arr2-WT+PP2	101 ± 4	5.4 ± 0.7	66 ± 12.6
Arr2-P91G/P121E+PP2	99 ± 2	4.0 ± 2.1	82 ± 6.8

^a^ All values represent mean +/- SEM.

### P91G/P121E arrestin mutant alters normal FPR trafficking and recycling

**Fig 2:**
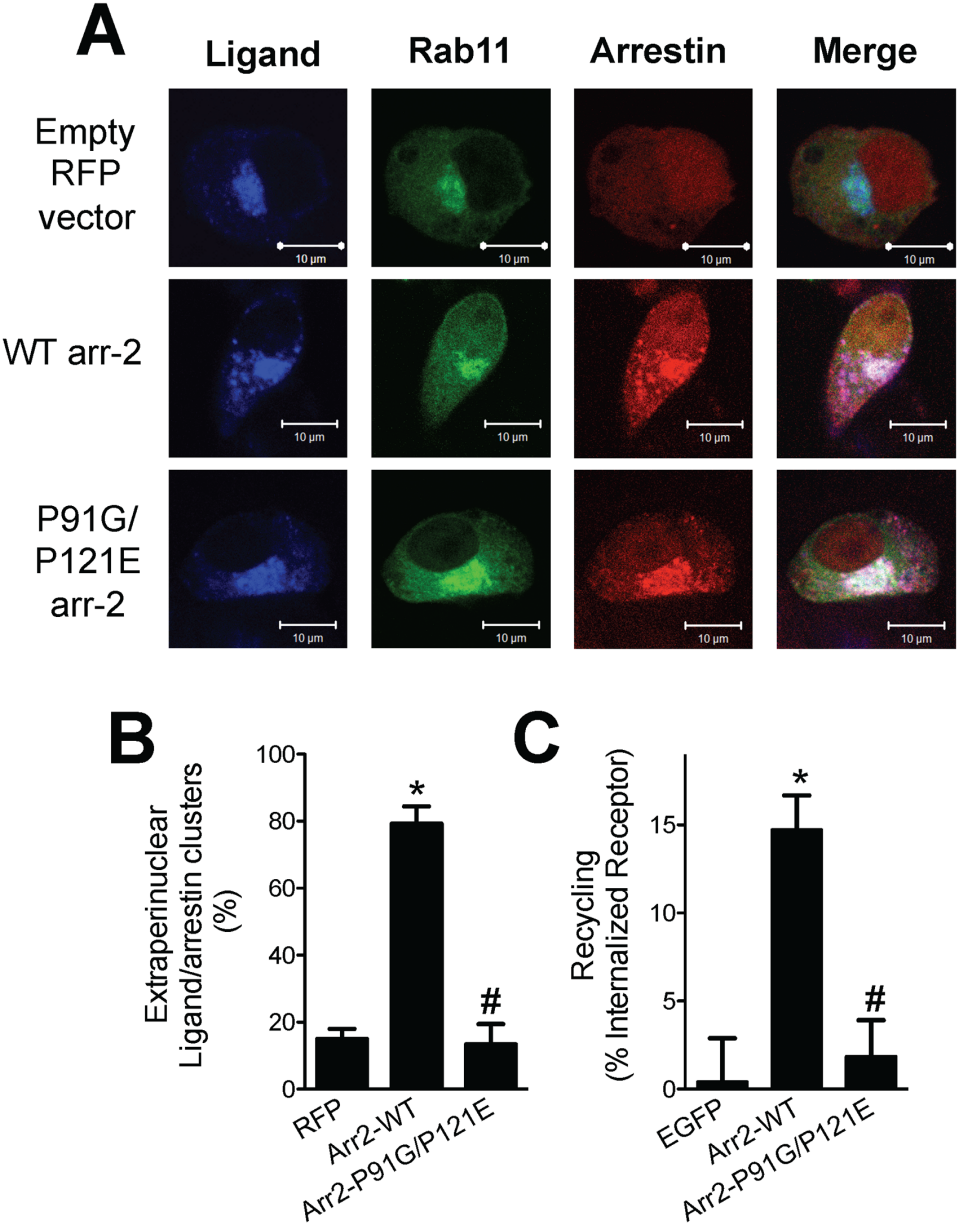
Defective FPR localization and recycling in the presence of arr2-P91G/P121E. **A)** Arr-2^-/-^/-3^-/-^ FPR cells were transiently co-transfected with GFP-fused Rab11 and RFP-fused arrestin constructs. Cells were stimulated with 10 nM 633-6pep for 1 hour and imaged by confocal fluorescence microscopy. Images are representative of three independent experiments. See Suppl. S1A Fig for unstimulated controls. **B)** Quantitation of the percent of cells displaying extraperinuclear (defined by perinuclear Rab11 localization) ligand-arrestin clusters. **C)** FPR recycling was assessed in arr-2^-/-^/-3^-/-^ FPR cells transfected with EGFP vector, arr2-WT-GFP or arr2-P91G/P121E-GFP. Data are expressed as mean recycling as a percentage of the internalized FPR +/- SEM from three independent experiments. * p<0.001 vs. RFP- (B) or p<0.01 vs. EGFP-transfected cells (C); # p<0.001 (B) or p<0.01 (C) vs. arr2-WT-transfected cells.

Since FPR-arrestin complex accumulation in the perinuclear endosomal sorting compartment correlates directly with FPR-mediated apoptosis [34], we hypothesized that the arr2-P91G/P121E mutant would follow such a paradigm. To test this, RFP-fused arrestins and GFP-fused Rab11 WT were transiently expressed in arr-2^-/-^/-3^-/-^ FPR MEF cells. The FPR was activated with the fluorescent ligand Alexa 633-*N*-formyl-Leucyl-Leucyl-Phenylalanyl-Leucyl-Tyrosinyl-Lysine (633-6pep) for 1 hour and ligand-FPR-arrestin complexes were visualized using confocal fluorescence microscopy (Fig 2A and S3A Fig). Additionally, we quantified the percentage of cells in which ligand-arrestin complexes were present in extraperinuclear (i.e. not colocalizing with Rab11-GFP) regions of the cytoplasm (Fig 2B). In unstimulated cells, both wild type and P91G/P121E arrestin were distributed throughout cells and Rab11 was observed in a perinuclear location (S1A Fig). Following stimulation, in the presence of mRFP1 vector alone (empty vector with no arrestin), the ligand-bound, internalized FPR (based on 633-6pep visualization) accumulated in perinuclear endosomes with few ligand-receptor-arrestin complexes present external to this region (Fig 2A). In the presence of arr2-WT-mRFP, ligand-receptor-arrestin complexes were localized both in the perinuclear endosomal recycling compartment and outside this region as puncta throughout the cytosol. These phenotypes are consistent with our previous results demonstrating the ability of the FPR to traffic normally in the presence of wild-type arrestin-2, but not in its absence [15,33,34]. In the presence of arr2-P91G/P121E-RFP, ligand-receptor-arrestin complexes accumulated predominantly in the perinuclear Rab11-GFP-positive endosomal compartment with very few endosomal ligand-receptor-arrestin complexes present outside the perinuclear region defined by Rab11. This is consistent with the inability of the FPR to recycle [15] and has been hypothesized as an underlying cause of FPR-mediated apoptosis in the absence of a functional wild type FPR-arrestin interaction [34]. Furthermore, these results also indicate that the arr2-P91G/P121E mutant binds to and traffics with the activated FPR. Direct measurements of FPR recycling confirm that as previously demonstrated [15], reconstitution with arr2-WT restores FPR recycling in arrestin-deficient cells; however, reconstitution with arr2-P91G/P121E does not support recycling (Fig 2C).

### Inhibition of Src kinase alters normal FPR trafficking

**Fig 3:**
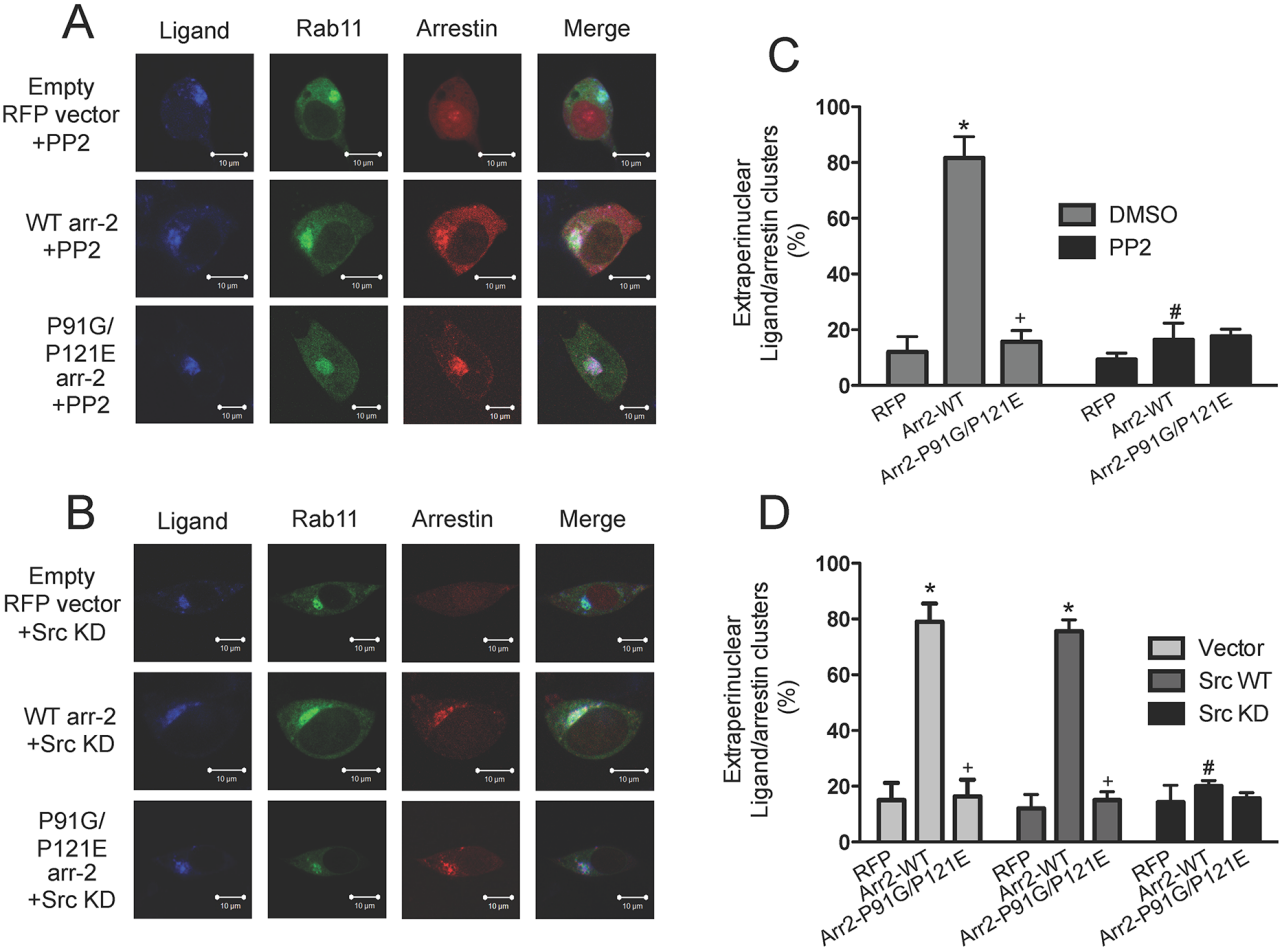
FPR-arrestin complexes exhibit differential trafficking in response to Src inhibition. Arr-2^-/-^/-3^-/-^ FPR cells were transiently transfected with the constructs indicated below. Cells were stimulated with 10 nM 633-6pep for 1 hour and imaged by confocal fluorescence microscopy. **A)** Arr-2^-/-^/-3^-/-^ FPR cells were transiently co-transfected with the indicated RFP-fused arrestin construct and GFP-fused Rab11 and incubated with PP2 (10 nM, 30 min) before and during ligand stimulation. See Suppl. S1B and S1C Fig for unstimulated controls and Suppl. S2A Fig for stimulated vehicle (no PP2) controls. **B)** Arr-2^-/-^/-3^-/-^ FPR cells were transiently transfected with GFP-fused Rab11, RFP-fused arrestins and kinase dead Src kinase. See Suppl. S1D–S1F Fig for unstimulated controls transfected with Src plasmid vector only (pUSE), wild type Src and kinase dead Src. See Suppl. S2B and S2C Fig for stimulated controls of cells transfected with Src plasmid vector only (pUSE) and wild type Src, respectively. Images are representative of three independent experiments. **C, D)** Quantitation of the percent of cells displaying extraperinuclear receptor ligand-arrestin complexes following treatment with PP2 (**C**) or transfection with kinase dead Src (**D**). Cells were counted from three independent experiments and data are expressed as the mean percentage ± SEM of cells displaying ligand-arrestin complexes outside the perinuclear region. Scale bars in images equal 10 μm. * p<0.001 vs. RFP-transfected cells; + p<0.001 vs. arr2-WT-transfected cells; # p<0.001 vs. corresponding DMSO-treated cells (C) or Src-WT-transfected cells (D) as appropriate.

Because arrestins play a critical role in normal post-endocytic FPR trafficking [32,34] and FPR-mediated apoptosis was inhibited by PP2 in the presence of arr2-P91G/P121E, we hypothesized that PP2 might restore normal FPR-arrestin trafficking. To test this, we used transfected arr-2^-/-^/-3^-/-^ FPR MEFs stimulated with ligand in the presence of either vehicle (S2A Fig) or PP2 (Fig 3A and 3C). In the absence of FPR stimulation, neither arrestin nor Rab11 localization was affected in the presence of vehicle (S1B Fig) or PP2 (S1C Fig), and PP2 demonstrated no effect on FPR trafficking in the absence of arrestins (mRFP1) as receptor continued to accumulate in recycling endosomes (Fig 3A and 3C). Additionally, inhibition of Src kinase activity with PP2 did not affect FPR trafficking in the presence of the P91G/P121E mutant as receptor-arrestin complexes continued to be retained in perinuclear endosomes. However, in the presence of wild type arrestin-2, PP2 led to the accumulation of the receptor-arrestin complex in the recycling endosome with few FPR-arrestin complexes found outside this region (Fig 3A and 3C). These results indicate that although the inhibition of Src kinase activity in the presence of a mutant arrestin with decreased binding to Src kinase can prevent apoptosis, it does not reverse the trafficking defects initiated by the Src-binding deficiency. Additionally, in the presence of WT arrestin, FPR activation in the presence of Src kinase inhibition alters receptor-arrestin trafficking similar to arr2-P91G/P121E or arrestin deficiency without initiating apoptosis (*cf*. Figs 3A and 1C).

To confirm that these alterations were specifically due to inhibition of Src kinase activity, receptor trafficking was also assessed in cells co-transfected with either wild type (S2C Fig) or kinase dead Src kinase (Fig 3B and 3D). Expression of wild type or kinase dead Src (or the empty pUSE vector) had no effect on arrestin or Rab11 localization in the absence of ligand stimulation (S1D–S1F Fig). Similarly, expression of wild type Src kinase or the empty vector had no effect on the trafficking of any of the receptor-arrestin complexes (S2B and S2C Fig). However, co-transfection of kinase dead Src kinase yielded similar results (Fig 3B and 3D) to the use of PP2 (Fig 3A and 3C) with virtually all ligand-FPR-arrestin complexes accumulating in recycling endosomes, thereby supporting the conclusion that the observed trafficking defects resulted from a lack of Src kinase activity.

### P91G/P121E arrestin mutant alters FPR interactions with adaptor protein complexes

**Fig 4:**
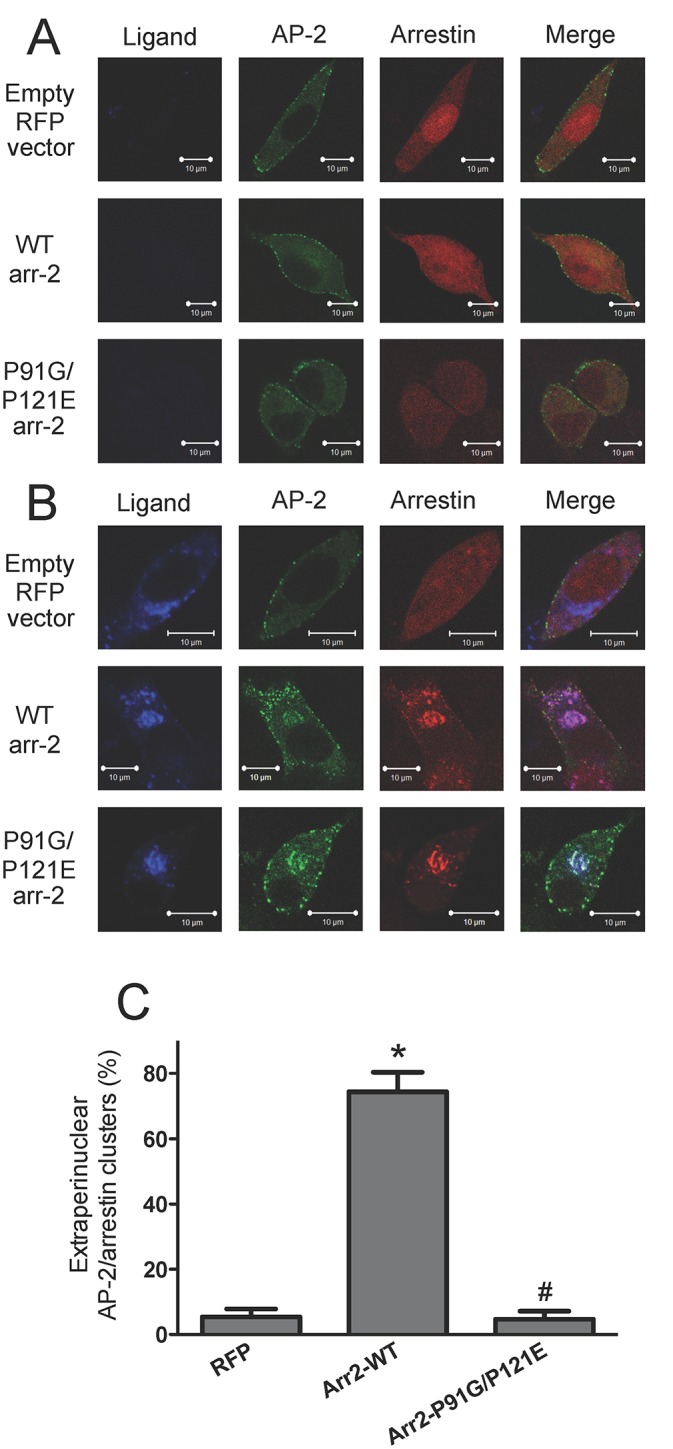
AP-2 accumulates in perinuclear endosomes with FPR-arrestin complexes in the absence of Src associating with arrestin. Arr-2^-/-^/-3^-/-^ FPR cells were transiently co-transfected with the indicated RFP-fused arrestins and the GFP-fused α-subunit of AP-2. Cells were either treated with vehicle (unstimulated) (**A**) or stimulated with 10 nM 633-6pep for 1 hour (**B**) and imaged by confocal fluorescence microscopy. Images are representative of three independent experiments. Scale bars equal 10 μm. **C)** Quantitation of the percentage of cells with extraperinuclear receptor ligand-arrestin/AP-2 complexes following ligand stimulation. Cells were counted from three independent experiments and data are expressed as the mean percentage ± SEM of cells displaying extraperinuclear colocalized ligand/arrestin/AP-2 clusters. * p<0.001 vs. RFP-transfected cells; # p<0.001 vs. arr2-WT-transfected cells.

Our previous results demonstrated a role for AP-2 in the post-endocytic trafficking of the FPR [34]. In that study, we concluded that AP-2 must bind and release arrestin to facilitate exit of the FPR from perinuclear endosomes and receptor recycling. Other reports have also demonstrated that Src kinase regulates arrestin-AP-2 interactions to control AT1_A_R internalization with Src kinase binding AP-2 and phosphorylating tyrosine residues of the β2-adaptin subunit in order for AP-2 to be released from arrestin-2 [12]. To better understand the role of Src kinase in the interaction of arrestin with AP-2, we utilized arr-2^-/-^/-3^-/-^ FPR MEFs stimulated with 633-6pep and transfected with the GFP-fused α-subunit of AP-2 and RFP-fused arrestins to monitor trafficking via confocal fluorescence microscopy (Fig 4 and S3B Fig). Antibody staining of the α-subunit of AP-2 previously confirmed that visualization of the α-GFP subunit represented endogenous AP-2 [34]. In unstimulated cells (Fig 4A), arrestins were distributed throughout cells with AP-2, which was also present in membrane-associated puncta, consistent with previous observations [34,37]. In the absence of arrestins, following a one-hour stimulation of the FPR with 633-6pep (Fig 4B), AP-2 failed to colocalize with the internalized FPR (in perinuclear endosomes), reflecting a requirement for arrestin to act as a scaffolding protein. In contrast, in the presence of arr2-WT, AP-2 colocalized with FPR and arr2-WT in the perinuclear region as well as in extraperinuclear puncta, as previously observed [34]. However, in the presence of the arr2-P91G/P121E mutant, AP-2 colocalized with the receptor-arrestin complex in the perinuclear region but not significantly in extraperinuclear locations, suggesting that despite the fact that this mutant arrestin exhibits decreased binding to Src kinase, initial arrestin-AP-2 binding occurs, but complexes cannot recycle becoming trapped in perinuclear endosomes (Fig 4B and 4C).

**Fig 5:**
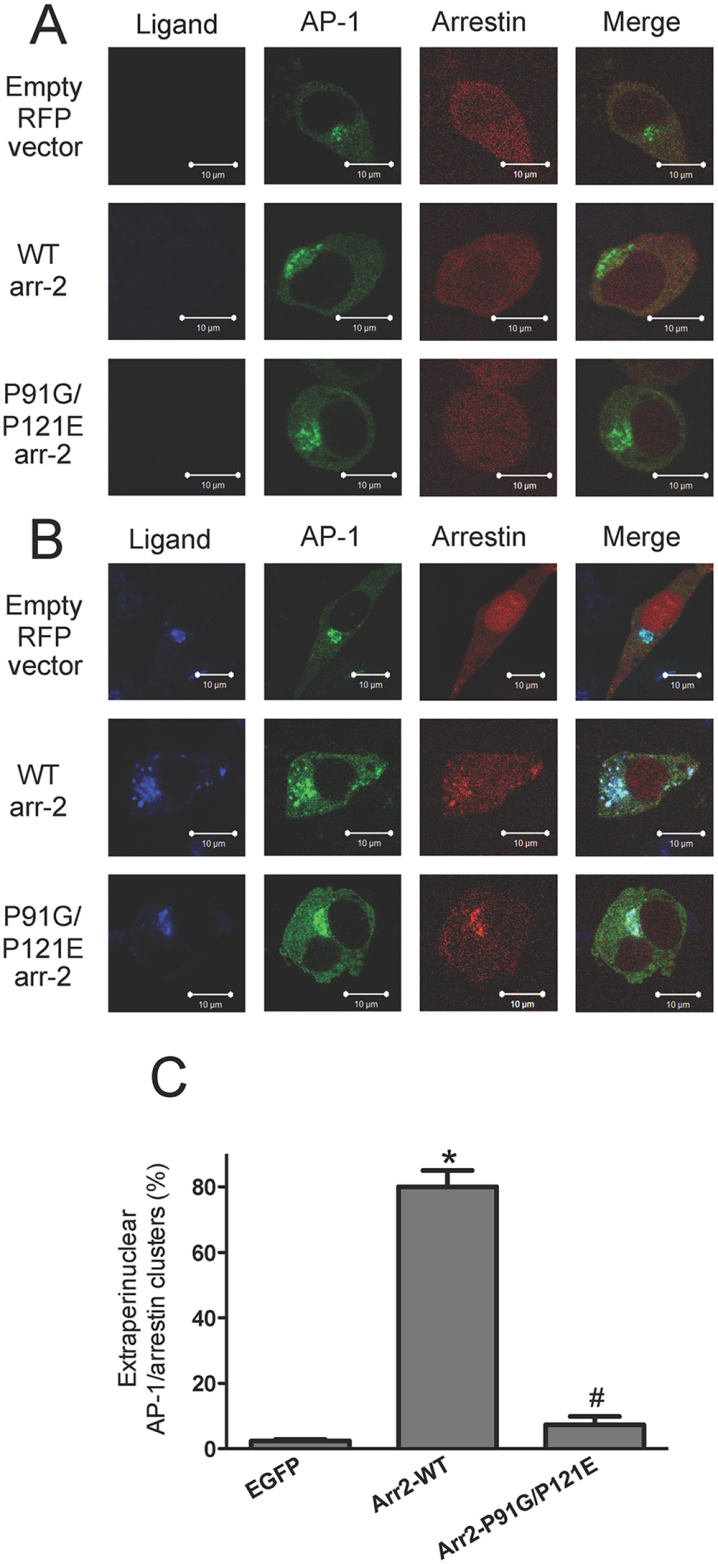
AP-1 accumulates in perinuclear endosomes with FPR-arrestin complexes in the absence of Src associating with arrestin. Arr-2^-/-^/-3^-/-^ FPR cells were transiently co-transfected with the indicated RFP-fused arrestins and the GFP-fused γ-subunit of AP-1. Cells were either treated with vehicle (unstimulated) (**A**) or stimulated with 10 nM 633-6pep for 1 hour (**B**). Transfected cells were imaged by confocal fluorescence microscopy. Images are representative of three independent experiments. Scale bars equal 10 μm. **C)** Quantitation of the percentage of cells with extraperinuclear receptor ligand-arrestin/AP-1 complexes following ligand stimulation. Cells were evaluated from three independent experiments and the results are expressed as the mean percentage ± SEM of cells displaying extraperinuclear colocalized ligand/arrestin/AP-1 clusters. * p<0.001 vs. EGFP-transfected cells; # p<0.001 vs. arr2-WT-transfected cells.

We have also shown that AP-1 colocalizes with receptor-associated vesicles outside the perinuclear region in the presence of wild type arrestin-2 [34]. However, in the absence of arrestins or the presence of an arrestin mutant that results in trafficking defects (eg. Arr2F391A), AP-1 remains localized to the perinuclear region [34]. Because FPR-arr2-P91G/P121E complexes accumulate in perinuclear recycling endosomes, we hypothesized that arr2-P91G/P121E would not colocalize with AP-1 vesicles outside of perinuclear recycling endosomes. To test this, we used arr-2^-/-^/-3^-/-^ FPR cells transfected with RFP-fused arrestins and the GFP-fused γ-subunit of AP-1 (Fig 5 and S3C Fig). Antibody staining of the γ-subunit of AP-1 has previously confirmed that localization of the γ-GFP subunit is similar to the localization of endogenous AP-1 [34]. In unstimulated cells, arrestins were cytosolic and AP-1 was predominantly localized in a perinuclear region (Fig 5A), consistent with our previous results [34]. Following stimulation for 1 hour with 633-6pep in the absence of arrestins (empty RFP vector), the FPR accumulated in AP-1-positive endosomes with virtually no FPR observed outside this compartment (Fig 5B). In the presence of Arr2-WT, receptor-arrestin complexes were also colocalized with AP-1 in a perinuclear region, but receptor-arrestin-AP-1 vesicles were also present throughout the cell. However, in ligand-stimulated cells, arr2-P91G/P121E colocalized with AP-1 and FPR in the perinuclear region but not in vesicles outside this region, consistent with the inhibition of FPR recycling from this compartment (Fig 5B and 5C).

### ERK1/2 signaling is required for Src-dependent FPR-mediated apoptosis

**Fig 6:**
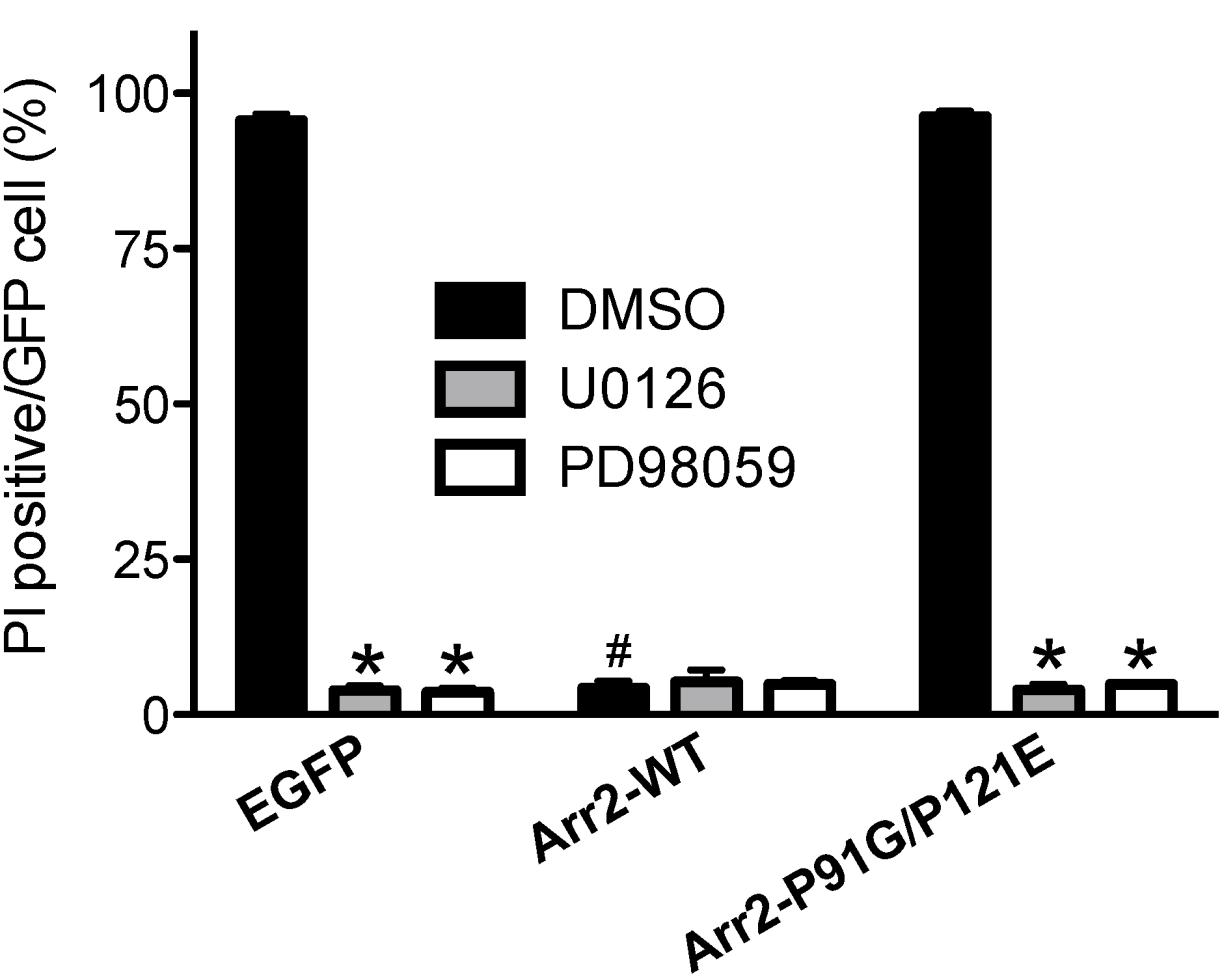
FPR-mediated apoptosis in the presence of arr2-P91G/P121E is sensitive to ERK inhibition. Arr-2^-/-^/-3^-/-^ FPR cells were transiently transfected with GFP-fused arrestins and pre-incubated with U0126 (10 μM, 30 min), PD98059 (25 μM, 30 min) or vehicle (DMSO). Cells were stimulated with 10 nM fMLF and stained with PI. Five random fields were viewed by fluorescence microscopy until 100–300 cells GFP cells were evaluated and GFP-expressing cells were scored for the presence of PI staining. Data are expressed as the mean PI positive/GFP cell +/- SEM from three independent experiments. * p<0.001 vs. corresponding DMSO-treated cells; # p<0.001 vs. EGFP-transfected cells.

While arr2-P91G/P121E displays decreased Src association and inhibits ERK1/2 activation in response to β2-AR activation, ERK1/2 activation in response to AT1_A_R activation is not similarly impaired [20]. In addition, GPCR-arrestin-Src scaffolds regulate localized activation of ERK1/2 and therefore other downstream processes [18,19,35]. Because Src has a clear role in FPR-mediated apoptosis (Fig 1) and because ERK1/2 inhibitors can inhibit FPR-mediated apoptosis in the absence of arrestins [33], we sought to determine the role of ERK1/2 with respect to apoptosis in the presence of the arr2-P91G/P121E mutant. To this end, we performed apoptosis assays using arr-2^-/-^/-3^-/-^ FPR cells transfected with GFP-fused arrestins in the presence of MAPK pathway inhibitors U0126 and PD98059 (Fig 6). In the presence of vehicle, apoptosis in cells transfected with GFP alone and arr2-P91G/P121E-GFP was greater than 90% and with wild type arrestin-2 was less than 5% consistent with previous data (Fig 1C). Interestingly, in the presence of MAPK pathway inhibitors U0126 or PD98059, transfected cells did not stain significantly with PI, indicating that MAPK pathway inhibition prevents FPR-mediated apoptosis in the absence of arrestins or in the presence of the P91G/P121E mutant, suggesting the possibility of common downstream signaling defects.

## Discussion

Receptor trafficking and signaling are intimately connected within the cell and their appropriate control is essential for normal cell function. We have previously demonstrated that these processes are linked as arrestin mutants that alter FPR signaling also alter its post-endocytic trafficking [33,34]. Our previous results have also suggested a role for Src kinase within these processes [33]. In our present study, we used an established arrestin mutant (P91G/P121E), previously shown to be defective in binding to Src kinase [20]. We demonstrated the mutant’s inability to prevent FPR-mediated apoptosis in the absence of endogenous arrestins, although cell death in the presence of this mutant was rescued by inhibition of Src kinase or ERK1/2 activity. Additionally, this mutant and Src kinase inhibition did not affect FPR internalization. Finally, our results indicated that FPR-arr2-P91G/P121E complexes accumulate in perinuclear recycling endosomes together with Rab11, AP-2 and AP-1. Although Src kinase inhibition rescued FPR-mediated apoptosis in the presence of arr2-P91G/P121E, it did not restore normal trafficking. Interestingly, Src kinase inhibition resulted in the accumulation of FPR-arr2-WT in the Rab11 endosomal compartment without initiating cell death. These results indicate that Src kinase has two independent roles with respect to the FPR-arrestin complex function: one that controls receptor trafficking and one that controls non-G protein-mediated receptor signaling.

The most intriguing results from our study involve the roles of Src kinase-arrestin interactions on FPR signaling. The P91G/P121E arrestin mutant (with deficient Src binding) did not rescue FPR-mediated apoptosis. However, inhibition of Src kinase and ERK1/2 activity in the presence of this mutant prevented cell death, indicating that proper arrestin-Src kinase binding is necessary to control non-G protein-mediated FPR signaling events. Furthermore, these data suggest that without arrestin regulation, Src and ERK1/2 signal aberrantly resulting in cell stress leading to apoptosis. Where and how these signaling proteins are being aberrantly activated by FPR stimulation is currently unclear and could involve arrestin-independent G protein-mediated activation of these pathways [38]. Alternatively, Src and ERK1/2 activation may result from arrestin-2 binding to discrete proteins not directly affected by Src kinase binding. For example, the ubiquitous signaling molecule calmodulin binds arrestins [39] and although the effects of this binding on GPCR signaling are currently unknown, they could theoretically indirectly activate the Src kinase pathway in an aberrant spatial or temporal manner.

Another interesting result from our study involves the role of arrestin-Src kinase interaction in FPR trafficking. The Src kinase binding-deficient arrestin mutant resulted in accumulation of FPR-arrestin complexes in the perinuclear region with Rab11, AP-2 and AP-1. This is perhaps not surprising given the fact that FPR-mediated apoptosis and FPR trafficking defects have been linked in previous work [34]. However, while Src kinase inhibition rescued FPR-mediated apoptosis, it did not restore normal FPR-arrestin trafficking. Furthermore, inhibition of Src kinase in the presence of wild type arrestin-2 caused FPR-arrestin complexes to accumulate in the perinuclear recycling endosome without initiating apoptosis. These results indicate a complex role for Src kinase in normal FPR post-endocytic trafficking. Previous reports have indicated that the Src-dependent phosphorylation of tyrosine residues on AP-2 is necessary to release AP-2 from arrestin and mediate internalization of the AT1_A_R, without which the receptor remains trapped at the cell surface in clathrin-coated pits [12]. Similarly, our previous results suggest that binding and release of AP-2 from arrestin-2 is necessary for proper post-endocytic trafficking of the FPR through recycling endosomes [34]. Our results show an accumulation of AP-2 with FPR-arrestin in perinuclear recycling endosomes in the absence of Src kinase activity. Therefore, we hypothesize in the absence of binding to arrestin-2 or inhibition of its activity, Src kinase cannot phosphorylate arrestin-bound AP-2 in the perinuclear recycling endosomal compartment, leading to accumulation of FPR-arrestin in this region, events that are independent of Src’s role in apoptotic signaling.

Finally, our previous results have shown that FPR internalization from the plasma membrane is not dependent on clathrin [40], AP-2 [34], or arrestin [15]. In this study, we further demonstrate that FPR internalization is independent of Src kinase. Our data demonstrate that the extent and rate of FPR internalization were not affected by an arrestin mutant that does not bind Src kinase (P91G/P121E) or by the use of the Src kinase family inhibitor PP2. Although the precise mechanisms of FPR internalization remain unclear, receptor phosphorylation by G protein-coupled receptor kinases is clearly required, as it is for receptor desensitization [41–44]. The patterns of FPR phosphorylation within the carboxy-terminus are very complex [45,46], with multiple effects on G protein and arrestin binding as well as ligand affinity [45,47–51], suggesting the existence of a multitude of receptor conformations that could lead to a plethora of distinct signaling and trafficking events [52].

**Fig 7:**
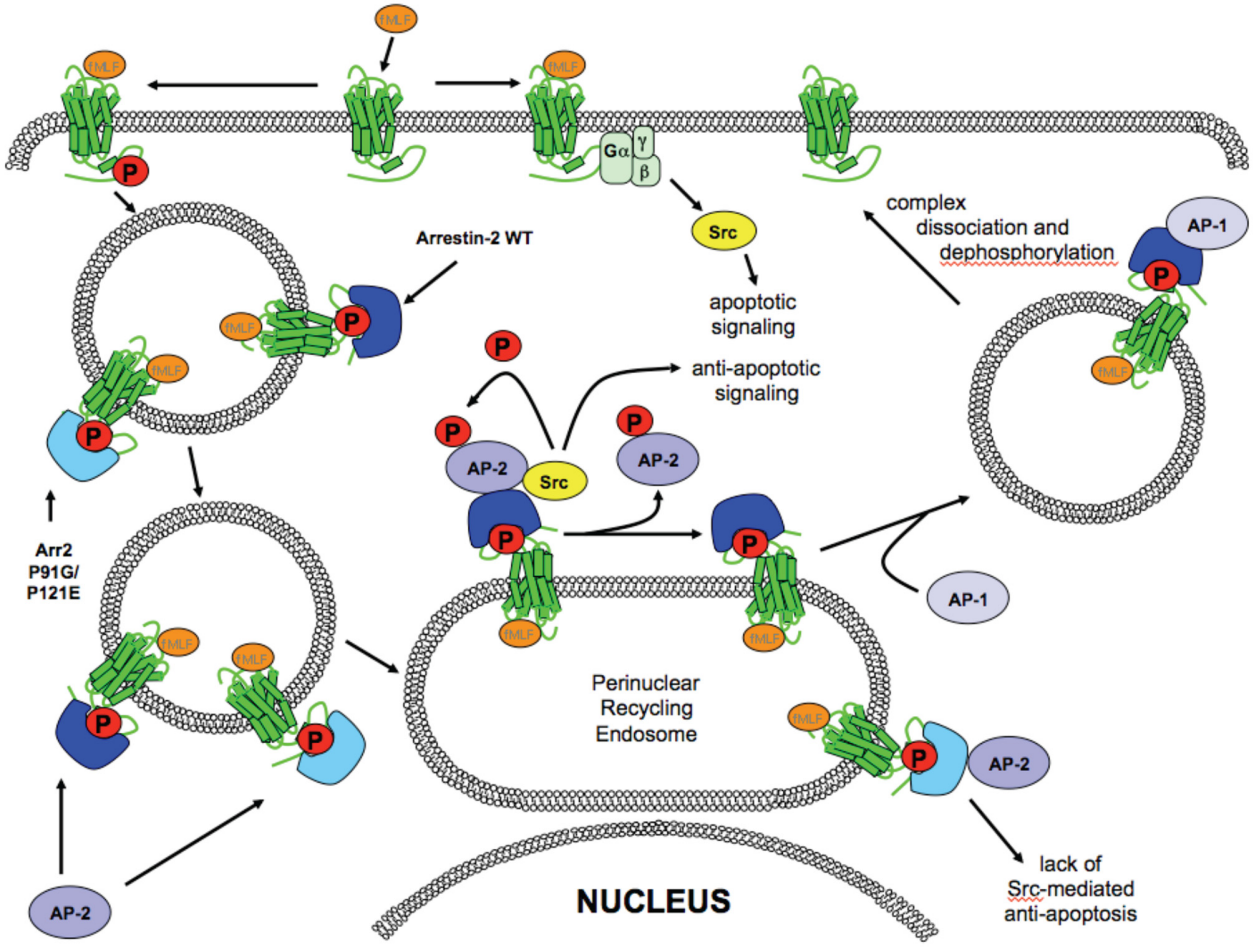
Model of FPR-mediated signaling, trafficking and apoptosis. See text for details.

Based on our results, we propose a model involving two independent roles for Src kinase in FPR trafficking and signaling (Fig 7). Our model is based upon a scheme of GPCR internalization from Ferguson and colleagues [53] and augmented with our current observations: Following ligand binding, the FPR activates G proteins, which, in turn, activate Src kinase-mediated apoptotic signaling pathways, which can be inhibited by G protein inhibition with pertussis toxin [33]. The FPR undergoes GRK-mediated phosphorylation and internalizes in an arrestin-independent manner. After, or perhaps during internalization, the FPR binds arrestin (wild type or P91G/P121E), resulting in the presence of FPR/arrestin complexes in early endosomes. This results in the desensitization of G protein-mediated signaling, potentially including arrestin-independent pro-apoptotic Src activation. At some point during its trafficking to Rab11 perinuclear recycling endosomes (or after its arrival at this location) the FPR-arrestin complex recruits AP-2. At this point (or perhaps earlier), Src kinase is recruited to the FPR-wild type arrestin-AP2 complex, which may also initiate spatially or temporally regulated anti-apoptotic signaling pathways to counter G protein-mediated apoptotic signaling. In addition, Src kinase phosphorylates AP-2 leading to its dissociation from arrestin followed by binding of AP-1. This sequence results in FPR-arrestin egress from perinuclear recycling endosomes. Along the path to the cell surface the complex dissociates, the receptor is dephosphorylated and completes its return to the cell surface in a resensitized form ready to continue signaling. Based on the studies reported here, we suggest that FPR-arr2-P91G/P121E-AP-2 complexes accumulate in the perinuclear recycling endosome due to the inability to bind Src kinase, which results in a lack of AP-2 phosphorylation. This, in turn, prevents Src kinase from initiating “anti-apoptotic signaling/activity” within the context of a GPCR-arrestin signaling scaffold, leaving the pro-apoptotic Src signaling to dominate cellular responses. This model suggests a highly complex array of spatially and temporally coordinated Src-mediated signaling events that are both pro-apoptotic and anti-apototic, with the ultimate cellular outcome dependent upon the integration of these events.

In conclusion, this report elucidates two independent roles for Src kinase in FPR trafficking and signaling. To our knowledge, this is the first indication that Src kinase has multiple roles within the same GPCR signaling complex: one in control of its trafficking and one in mediating proper cellular signaling. Both of these roles are coordinated and regulated by binding to arrestin-2. Our results also clarify a role for arrestin-2 in the spatial and temporal control of non-G protein-mediated GPCR signaling and serve as a model for further study. Consistent with our results, recent reports also indicate a role for AP-2 in the apoptosis mediated by visual arrestin binding to a rhodopsin mutant found in certain humans diagnosed with autosomal dominant retinitis pigmentosa [54]. Arrestins have also been shown to regulate apoptosis following serum deprivation through the regulation of ERK1/2, p38 and Akt pathways [55]. Roles for arrestins, Src kinase, ERK1/2, adaptor proteins and the ubiquitination machinery have also been implicated in the post-endocytic sorting of chemokine, thrombin and other GPCRs [56–60]. An interesting question that arises from this work is whether trafficking defects alter cell signaling or vice-versa? With this report of novel roles for arrestins and Src kinase in the trafficking and signaling of GPCRs, new avenues for the targeting of GPCR function are presented that may lead to therapeutic interventions for disease processes through the induction or inhibition of cellular apoptosis.

## Materials and Methods

### Materials, Plasmids and Mutagenesis

All materials were from Sigma unless otherwise specified. Arr2-P91G/P121E-GFP was created by using a two-step PCR mutagenesis procedure. Briefly, primer pairs 5’-GCCCATATGGGCGACAAAGGGACGCGG-3’ and 5’-GCCCAGCTTCTTGATGAGGCGCTCCTGCAGCCGCGTCAGGGGCTTCTTGTCCTCACCGGCCGGCGGGAAAGACTGCACG-3’ and primer pairs 5’-GCAGGAGCGCCTCATCAAGAAGCTGGGCGAGCATGCCTACCCTTTCACCTTTGAGATCCCTGAGAACTCCCATGCTCTGTGACTTTGCAGCCG-3’ and 5’-GGATCCCGGGCCCATCTGTCGTTGAGCCGCGG-3’ were used to create N- and C-terminal fragments that contained the P91G and P121E mutations, respectively. Fragments were purified and amplified using the external primers to create an Arr2-P91G/P121E fragment containing HindIII/ApaI restriction sites. Standard subcloning methods were used to insert this fragment into the EGFP or mRFP1 vector. The presence of the correct sequence was verified by Sanger dideoxy sequencing of the entire coding sequence. Other constructs, including mRFP1, Arr2-WT-RFP, EGFP, Arr2-WT-GFP, Rab11-GFP, γ-subunit (AP-1)-GFP and α-subunit (AP-2)-GFP were previously described [34]. Murine wild type and kinase dead Src kinase constructs in pUSE, as well as the empty pUSE vector (Upstate Cell Signaling Solutions), were a gift from Steve Abcouwer.

### Cell Culture and Transfection

Arr-2^-/-^/-3^-/-^ FPR cells were grown in Dulbecco’s Modified Eagle Medium (DMEM) with 10% fetal bovine serum, 100 units/mL penicillin and 100units/mL streptomycin at 37°C and 5% CO_2_. Transient transfections of arr-2^-/-^/-3^-/-^ FPR cells were performed with Lipofectamine 2000 according to manufacturer’s instructions.

### Apoptosis

Apoptosis assay was performed as previously described [33]. Briefly, arr-2^-/-^/-3^-/-^ FPR cells were transiently transfected and plated on 12mm glass coverslips. The next day, cells were serum-starved for 30 min and incubated with serum-free medium (SFM) for 5 hours with 10 nM formyl-methionyl-leucyl-phenylalnine (fMLF) or vehicle at 37°C. Propidium iodide (PI) was then added to a final concentration of 100 pg/μL for 5–10 min at room temperature. Coverslips were washed and fixed with 2% paraformaldehyde and mounted using Vectashield. Random fields were viewed by fluorescence microscopy until 100–300 GFP expressing cells were assessed. GFP cells were scored for the presence of PI staining. Data are expressed as mean PI positive/GFP cell.

### Receptor Internalization

Internalization was performed as previously described [15]. Briefly, transiently transfected arr-2^-/-^/-3^-/-^ FPR cells were grown to confluence and harvested by trypsinization, which does not affect FPR binding of ligand (comparing non-trypsinized to trypsinized cells, the latter exhibit 98+/-6% the binding levels of the former (n = 3, p = ns). Cells were incubated with 1 μM fMLF at 37°C and aliquots were removed and placed in cold SFM at 0, 2, 5, 10, 20 and 30 min. Cells were washed extensively with cold SFM to remove excess fMLF. Cells were then resuspended in cold SFM containing 10 nM Alexa633-*N*-formyl-Leucyl-Leucyl-Phenylalanyl-Leucyl-Tyrosinyl-Lysine (633-6pep) and analyzed using a Becton Dickinson FACSCalibur at appropriate wavelengths. Live cells were gated using forward- and side-scatter parameters. Cells expressing the GFP-fused protein of interest were gated using FL-1 and mean channel fluorescence (MCF) was measured in FL-4 to determine amount of cell surface receptor remaining. Non-specific binding was determined by labeling arr-2^-/-^/-3^-/-^ cells not expressing the FPR with 10 nM 633-6pep and was subtracted before further analysis. MCF from unstimulated cells represents 100% FPR cell surface expression with non-specific binding representing less than 3–5% of the signal in the presence of FPR expression. Cell surface expression from stimulated cells was calculated by dividing the MCF following treatment by the MCF from unstimulated cells. Internalization data were then plotted using GraphPad Prism to calculate maximum internalization using a one phase exponential decay.

### Receptor Recycling

FPR recycling assays were performed as previously described [15]. Briefly, arr2^-/-^/3^-/-^ FPR cells transiently transfected with GFP-fused arrestins were harvested and resuspended in SFM. An aliquot was removed to measure total cell surface receptor. The remaining cells were stimulated with 1μM fMLF in SFM at 37°C for 1 hour and were then washed extensively to remove excess unlabelled ligand. Half the remaining cells were resuspended in pre-warmed SFM for 30 min at 37°C to allow the FPR to recycle. The other half was kept on ice to measure post-internalization cell surface receptor levels. All aliquots were then resuspended in SFM containing 10nM 633-6pep and assayed by flow cytometry using a Becton-Dickinson FACSCalibur. For analysis, cells were first gated for live cells using forward and side scatter parameters and then gated using FL-1 for GFP-fused arrestin mutant expression with the mean channel fluorescence measured in FL-4 to determine cell surface expression of the FPR. Non-specific binding was determined by labeling arr2^-/-^/3^-/-^ MEF cells (not expressing the FPR) with 10nM 633-6pep and subtracting this value from FPR-expressing cell values. To account for differences in absolute recycling potentially due to differences in the initial extent of internalization, the fraction of recycled FPR (starting from the final internalization time point) was divided by the fraction of internalized FPR. Data are expressed as a percentage of recycled/internalized receptor.

### Confocal Fluorescence Microscopy

Microscopy was performed as previously described [15]. Briefly, arr-2^-/-^/-3^-/-^ FPR cells were transiently transfected with the appropriate plasmids. Cells were plated on 25 mm coverslips, grown overnight and serum-starved for 30 min. Cells were then incubated in SFM containing 10 nM 633-6pep for the indicated times, washed with PBS, fixed with 2% paraformaldehyde and mounted using Vectashield. Fluorescence images were acquired using a Zeiss LSM 510 inverted laser scanning microscope equipped with He-Ne and Kr-AR lasers. To assess ligand/arrestin clusters outside the perinuclear region, cells were viewed and scored for the presence of ligand/arrestin outside the perinuclear Rab11-GFP-defined region in a blinded manner. Cells with less than 5 clusters outside of the Rab-11-GFP region were considered to exhibit accumulation without trafficking. To assess the extent of AP-2 or AP-1 colocalization, cells with arrestin clusters were viewed and scored for the presence of any corresponding AP-2 or AP-1 puncta, respectively. Data were expressed as the percentage of cells (mean ± SEM) with colocalized clusters from more than 25 cells/experiment.

## Supporting Information

S1 Fig Lack of arrestin and Rab11 colocalization in unstimulated Arr-2^-/-^/-3^-/-^ FPR cells. Arr-2^-/-^/-3^-/-^ FPR cells were transiently co-transfected with Rab11-GFP and either empty mRFP vector (Empty), wild type arrestin-2-RFP (WT) or arr2-P91G/P121E-RFP (P91G/P121E) along with the pUSE Src construct indicated below (D-F). Cells were treated and processed as described for ligand stimulation but in the absence of added ligand (resulting in no blue ligand signal). ***A)*** No treatment, ***B)*** DMSO (PP2 vehicle), ***C)*** PP2, ***D)*** pUSE vector only (pUSE), ***E)*** wild type Src (Src WT) and ***F)*** kinase dead Src (Src KD). All images demonstrate a lack of arrestin colocalization with Rab11. Scale bars equal 10μm. Images are representative of three independent experiments.
(PDF)

S2 Fig Ligand, arrestin and Rab11 localization in stimulated Arr-2^-/-^/-3^-/-^ FPR cells. Rab11-GFP and either empty mRFP vector (Empty), wild type arrestin-2-RFP (WT) or arr2-P91G/P121E-RFP (P91G/P121E) along with the pUSE Src construct indicated below (B-C). Cells were stimulated with 10 nM 633-6pep for 60 min and viewed by confocal fluorescence microscopy. ***A)*** Arr-2^-/-^/-3^-/-^ FPR cells were transiently transfected with RFP-fused arrestins (or vector only) and GFP-fused Rab11. Cells were incubated with DMSO (vehicle) for 30 min before and during stimulation as a control for PP2 treatment (see Fig 3A). See Fig 3D for quantitation. ***B)*** Arr-2^-/-^/-3^-/-^ FPR cells were transiently transfected with RFP-fused arrestins and GFP-fused Rab11 and the pUSE empty vector as a control for wild type (see **C** below) and kinase dead Src (see Fig 3B). See Fig 3C for quantitation. ***C)*** Arr-2^-/-^/-3^-/-^ FPR cells were transiently transfected with GFP-fused Rab11, RFP-fused arrestins and wild type Src kinase in pUSE as a control for kinase dead Src (see Fig 3B). See Fig 3D for quantitation. Scale bars equal 10μm. Images are representative of three independent experiments.
(PDF)

S3 Fig Line scans of ligand, arrestin and either Rab11, AP-2 or AP-1 demonstrating colocalization in 633-6pep stimulated Arr-2^-/-^/-3^-/-^ FPR cells. Arr-2^-/-^/-3^-/-^ FPR cells were transiently co-transfected with either Rab11-GFP ***(A)***, AP-2-GFP ***(B)*** or AP-1-GFP ***(C)*** and either empty mRFP vector (mRFP only), wild type arrestin-2-RFP (Arrestin-WT) or arr2-P91G/P121E-RFP (Arrestin-P91G/P121E). Cells were stimulated with 10 nM 633-6pep for 60 min and viewed by confocal fluorescence microscopy, followed by line intensity scanning in Zen software. Images are representative of three independent experiments.
(PDF)
