# Supplementary material for: Regulation of N-Formyl Peptide Receptor Signaling and Trafficking by Arrestin-Src Kinase Interaction
Source: PLoS One. 2016 Jan 20;11(1):e0147442. doi: 10.1371/journal.pone.0147442 (PMC4720441; doi:10.1371/journal.pone.0147442)
Supplement: S1 Fig — Arr-2-/-/-3-/- FPR cells were transiently co-transfected with Rab11-GFP and either empty mRFP vector (Empty), wild type arrestin-2-RFP (WT) or arr2-P91G/P121E-RFP (P91G/P121E) along with the pUSE Src construct indicated below (D-F). Cells were treated and processed as described for ligand stimulation but in the absence of added ligand (resulting in no blue ligand signal). A) No treatment, B) DMSO (PP2 vehicle), C) PP2, D) pUSE vector only (pUSE), E) wild type Src (Src WT) and F) kinase dead Src (Src KD). All images demonstrate a lack of arrestin colocalization with Rab11. Scale bars equal 10μm. Images are representative of three independent experiments. (PDF) [file pone.0147442.s001.pdf]

Supporting Information S1 Fig

Regulation of *N*-formyl Peptide Receptor Signaling and Trafficking by Arrestin-Src Kinase Interaction

Brant M. Wagener, Nicole A. Marjon and Eric R. Prossnitz

S1 Fig. Lack of arrestin and Rab11 colocalization in unstimulated Arr-2$^{-/-}$/-3$^{-/-}$ FPR cells. Arr-2$^{-/-}$/-3$^{-/-}$ FPR cells were transiently co-transfected with Rab11-GFP and either empty mRFP vector (Empty), wild type arrestin-2-RFP (WT) or arr2-P91G/P121E-RFP (P91G/P121E) along with the pUSE Src construct indicated below (D-F). Cells were treated and processed as described for ligand stimulation but in the absence of added ligand (resulting in no blue ligand signal). ***A)*** No treatment, ***B)*** DMSO (PP2 vehicle), ***C)*** PP2, ***D)*** pUSE vector only (pUSE), ***E)*** wild type Src (Src WT) and ***F)*** kinase dead Src (Src KD). All images demonstrate a lack of arrestin colocalization with Rab11. Scale bars equal 10 μm. Images are representative of three independent experiments.

S1 Fig

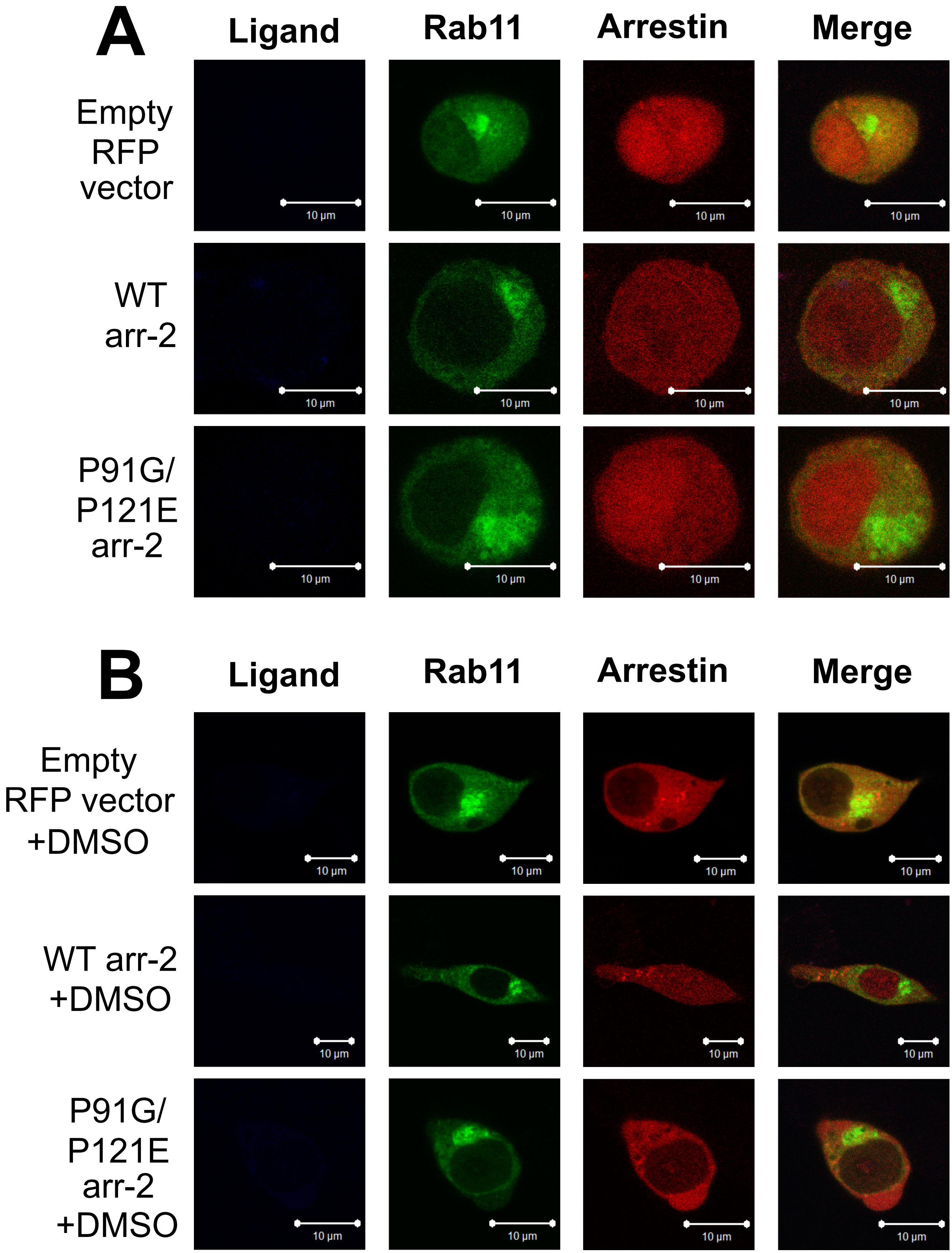
A
Ligand
Rab11
Arrestin
Merge
Empty RFP vector
WT arr-2
P91G/ P121E arr-2
10 µm
B
Ligand
Rab11
Arrestin
Merge
Empty RFP vector +DMSO
WT arr-2 +DMSO
P91G/ P121E arr-2 +DMSO
10 µm

S1 Fig

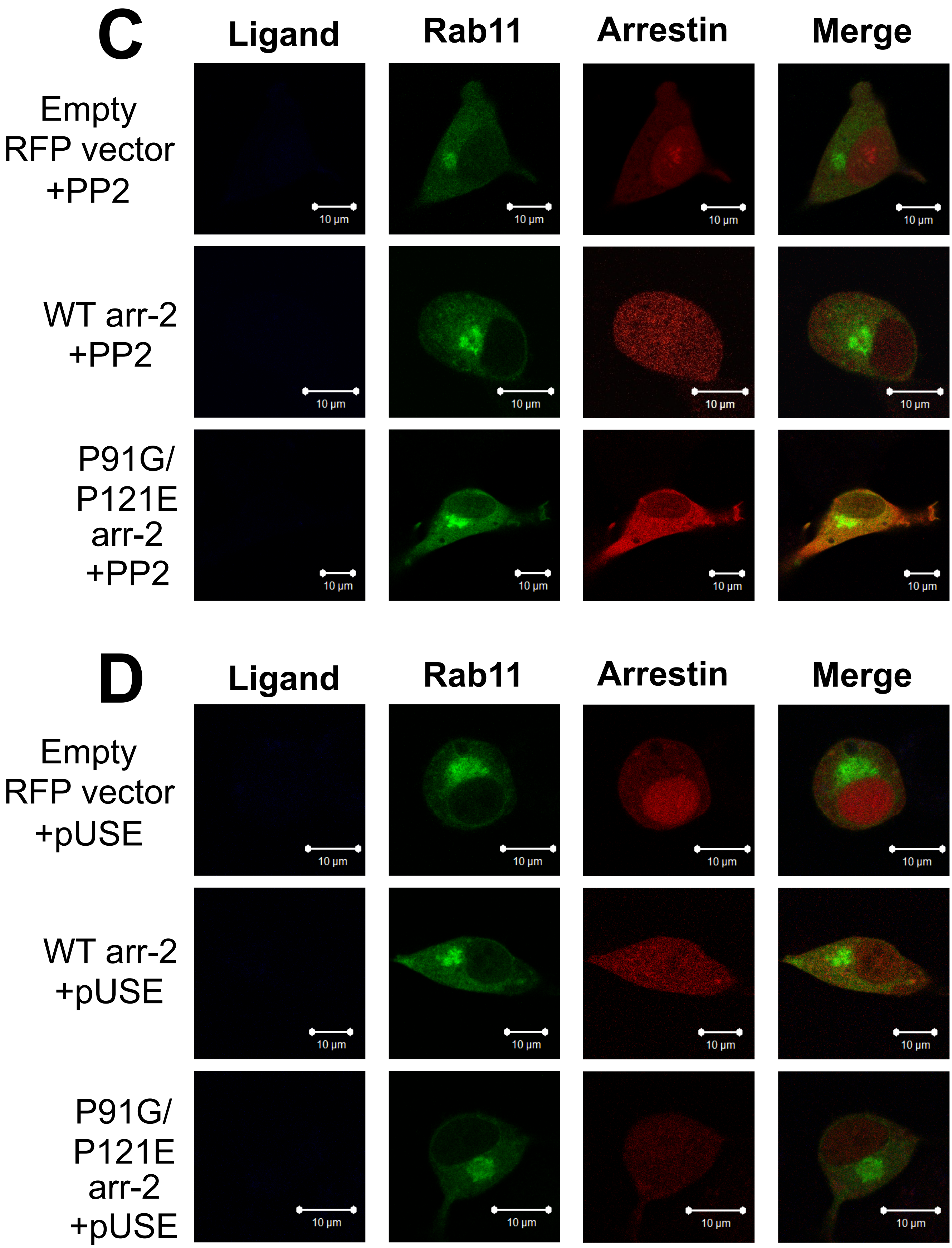
C
Ligand
Rab11
Arrestin
Merge
Empty RFP vector +PP2
WT arr-2 +PP2
P91G/ P121E arr-2 +PP2
10 μm
D
Ligand
Rab11
Arrestin
Merge
Empty RFP vector +pUSE
WT arr-2 +pUSE
P91G/ P121E arr-2 +pUSE
10 μm

S1 Fig

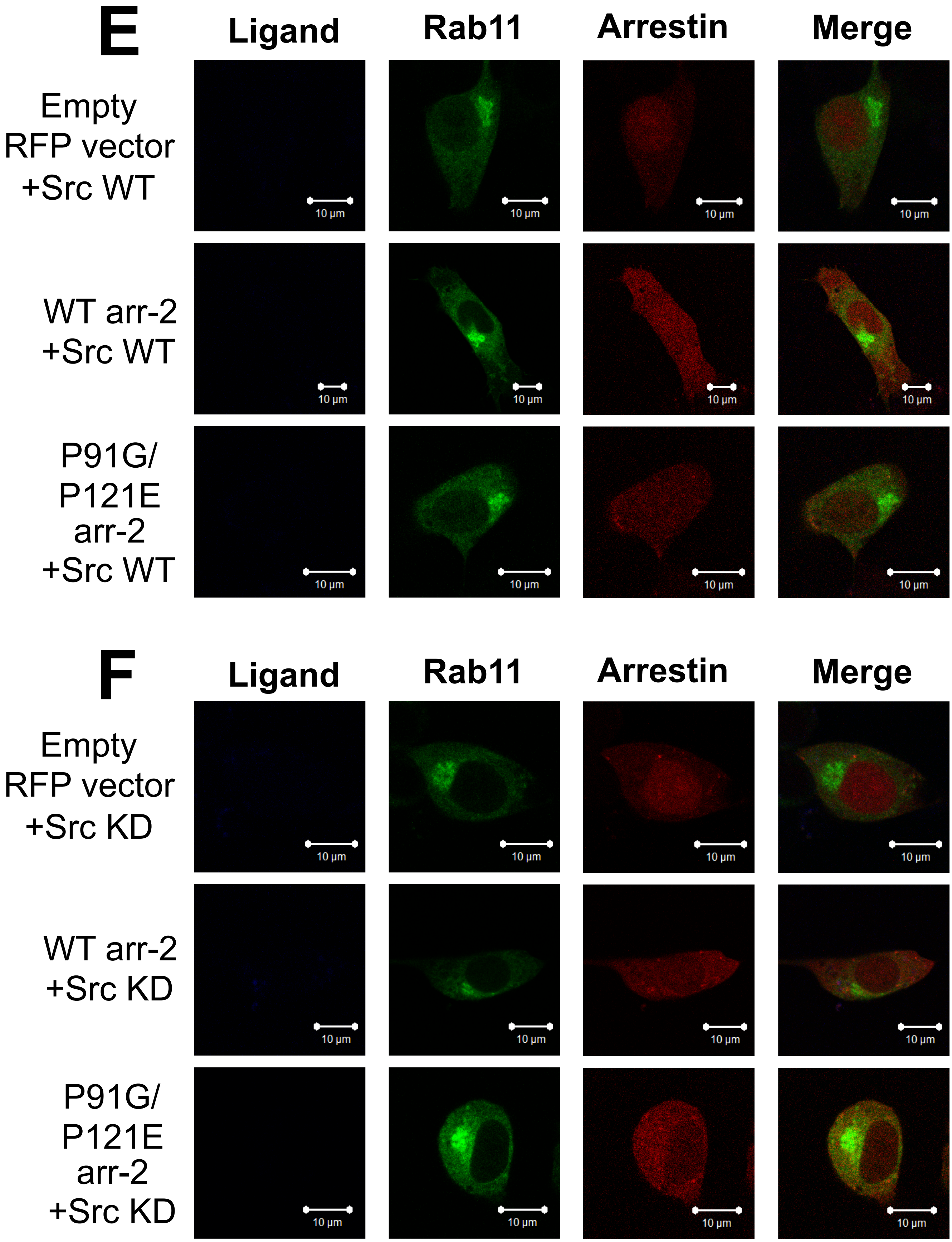
E
Ligand
Rab11
Arrestin
Merge
Empty RFP vector +Src WT
WT arr-2 +Src WT
P91G/ P121E arr-2 +Src WT
10 µm
F
Ligand
Rab11
Arrestin
Merge
Empty RFP vector +Src KD
WT arr-2 +Src KD
P91G/ P121E arr-2 +Src KD
10 µm
