# Supplementary material for: Regulation of N-Formyl Peptide Receptor Signaling and Trafficking by Arrestin-Src Kinase Interaction
Source: PLoS One. 2016 Jan 20;11(1):e0147442. doi: 10.1371/journal.pone.0147442 (PMC4720441; doi:10.1371/journal.pone.0147442)
Supplement: S2 Fig — Rab11-GFP and either empty mRFP vector (Empty), wild type arrestin-2-RFP (WT) or arr2-P91G/P121E-RFP (P91G/P121E) along with the pUSE Src construct indicated below (B-C). Cells were stimulated with 10 nM 633-6pep for 60 min and viewed by confocal fluorescence microscopy. A) Arr-2-/-/-3-/- FPR cells were transiently transfected with RFP-fused arrestins (or vector only) and GFP-fused Rab11. Cells were incubated with DMSO (vehicle) for 30 min before and during stimulation as a control for PP2 treatment (see Fig 3A). See Fig 3D for quantitation. B) Arr-2-/-/-3-/- FPR cells were transiently transfected with RFP-fused arrestins and GFP-fused Rab11 and the pUSE empty vector as a control for wild type (see C below) and kinase dead Src (see Fig 3B). See Fig 3C for quantitation. C) Arr-2-/-/-3-/- FPR cells were transiently transfected with GFP-fused Rab11, RFP-fused arrestins and wild type Src kinase in pUSE as a control for kinase dead Src (see Fig 3B). See Fig 3D for quantitation. Scale bars equal 10μm. Images are representative of three independent experiments. (PDF) [file pone.0147442.s002.pdf]

Supporting Information S2 Fig

Regulation of *N*-formyl Peptide Receptor Signaling and Trafficking by Arrestin-Src Kinase Interaction

Brant M. Wagener, Nicole A. Marjon and Eric R. Prossnitz

S2 Fig. Ligand, arrestin and Rab11 localization in stimulated Arr-2$^{-/-}$/-3$^{-/-}$ FPR cells. Arr-2$^{-/-}$/-3$^{-/-}$ FPR cells were transiently co-transfected with Rab11-GFP and either empty mRFP vector (Empty), wild type arrestin-2-RFP (WT) or arr2-P91G/P121E-RFP (P91G/P121E) along with the pUSE Src construct indicated below (B-C). Cells were stimulated with 10 nM 633-6pep for 1 hour and viewed by confocal fluorescence microscopy. ***A)*** Arr-2$^{-/-}$/-3$^{-/-}$ FPR cells were transiently transfected with RFP-fused arrestins (or vector only) and GFP-fused Rab11. Cells were incubated with DMSO (vehicle) for 30 min before and during stimulation as a control for PP2 treatment (see Figure 3A). See Figure 3D for quantitation. ***B)*** Arr-2$^{-/-}$/-3$^{-/-}$ FPR cells were transiently transfected with RFP-fused arrestins and GFP-fused Rab11 and the pUSE empty vector as a control for wild type (see **C** below) and kinase dead Src (see Figure 3B). See Figure 3C for quantitation. ***C)*** Arr-2$^{-/-}$/-3$^{-/-}$ FPR cells were transiently transfected with GFP-fused Rab11, RFP-fused arrestins and wild type Src kinase in pUSE as a control for kinase dead Src (see Figure 3B). See Figure 3D for quantitation. Scale bars equal 10 µm. Images are representative of three independent experiments.

S2 Fig

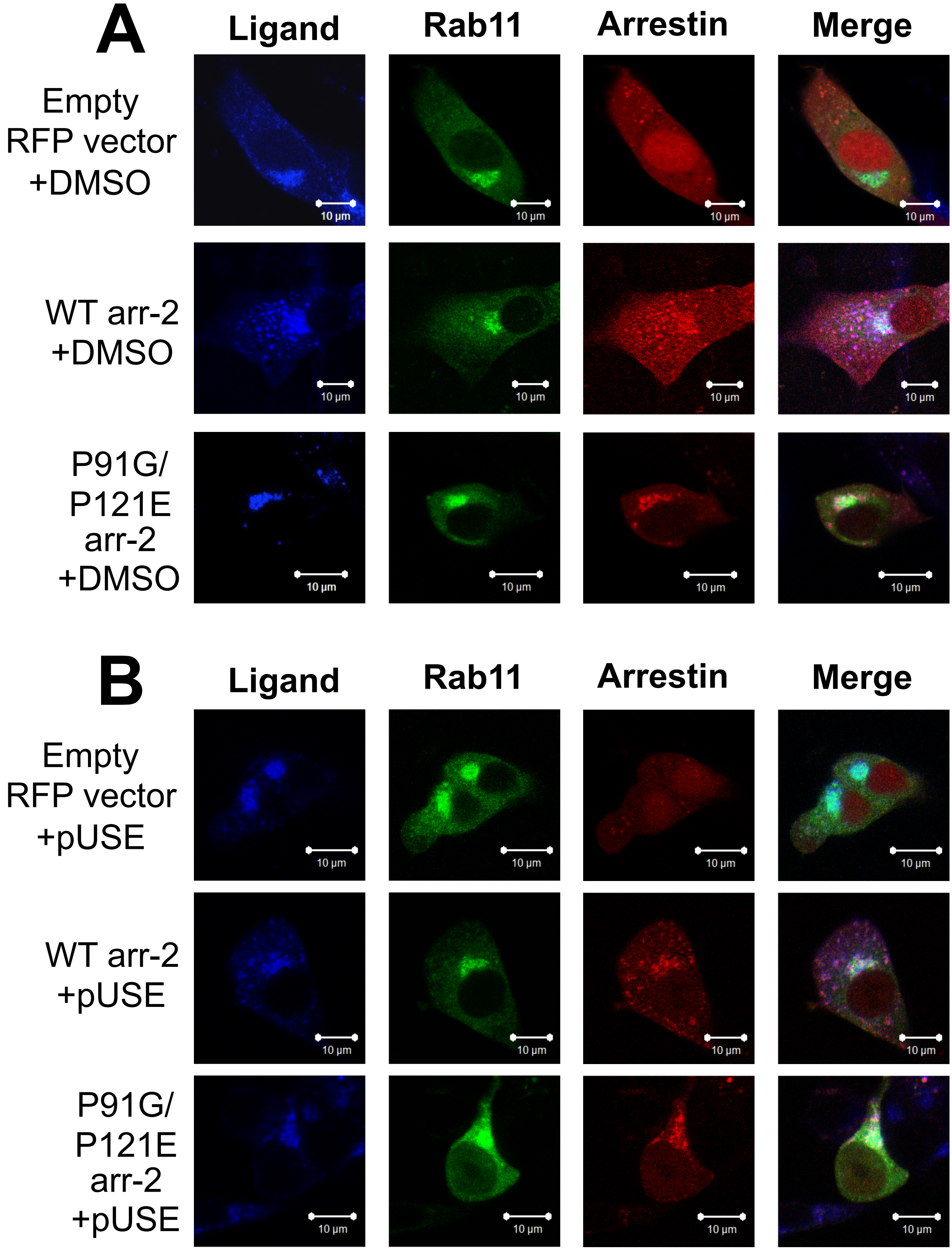
A
Ligand
Rab11
Arrestin
Merge
Empty RFP vector +DMSO
WT arr-2 +DMSO
P91G/ P121E arr-2 +DMSO
B
Ligand
Rab11
Arrestin
Merge
Empty RFP vector +pUSE
WT arr-2 +pUSE
P91G/ P121E arr-2 +pUSE
10 µm

S2 Fig

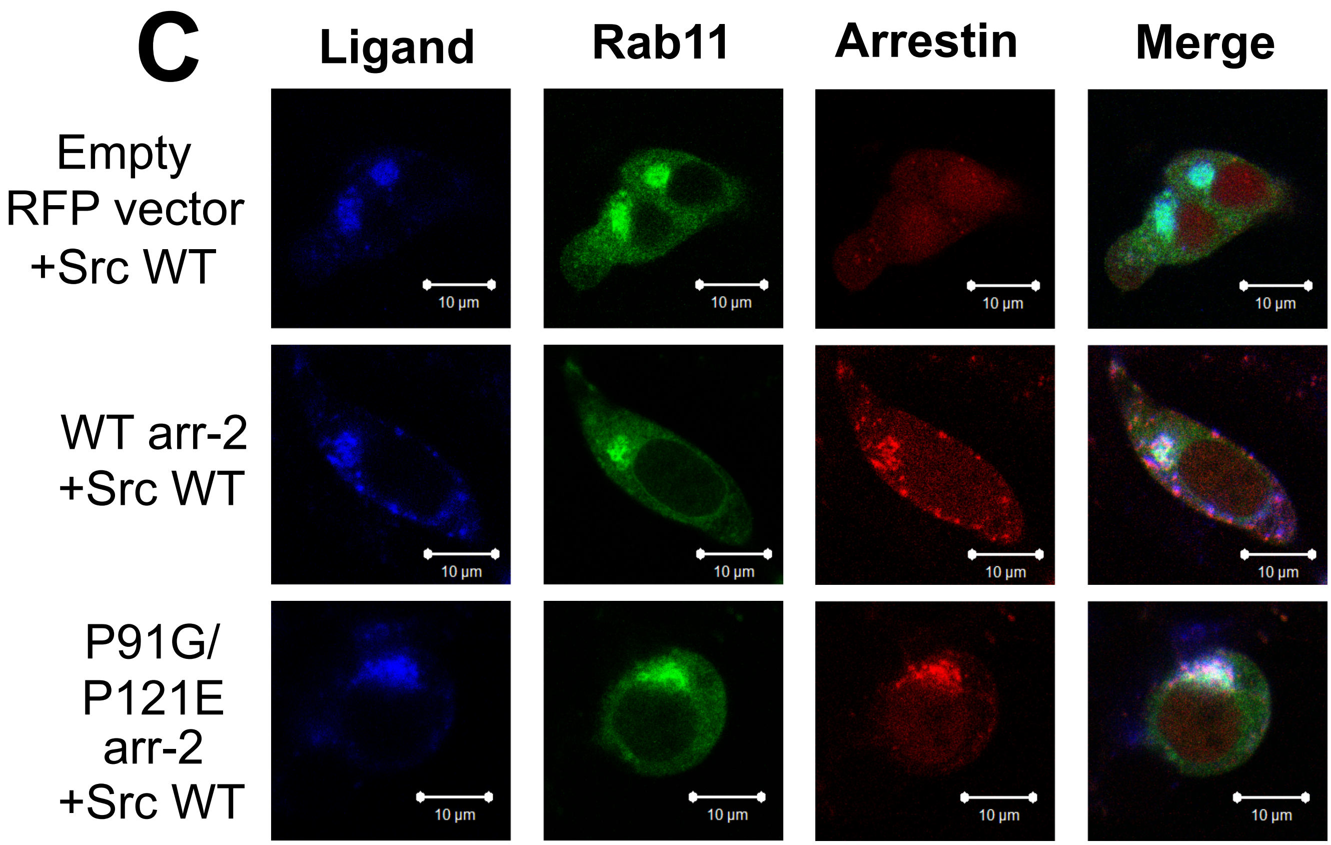
C
Ligand
Rab11
Arrestin
Merge
Empty RFP vector +Src WT
WT arr-2 +Src WT
P91G/ P121E arr-2 +Src WT
10 µm
