# Supplementary material for: Regulation of N-Formyl Peptide Receptor Signaling and Trafficking by Arrestin-Src Kinase Interaction
Source: PLoS One. 2016 Jan 20;11(1):e0147442. doi: 10.1371/journal.pone.0147442 (PMC4720441; doi:10.1371/journal.pone.0147442)
Supplement: S3 Fig — Arr-2-/-/-3-/- FPR cells were transiently co-transfected with either Rab11-GFP (A), AP-2-GFP (B) or AP-1-GFP (C) and either empty mRFP vector (mRFP only), wild type arrestin-2-RFP (Arrestin-WT) or arr2-P91G/P121E-RFP (Arrestin-P91G/P121E). Cells were stimulated with 10 nM 633-6pep for 60 min and viewed by confocal fluorescence microscopy, followed by line intensity scanning in Zen software. Images are representative of three independent experiments. (PDF) [file pone.0147442.s003.pdf]

Supporting Information S3 Fig

Regulation of *N*-formyl Peptide Receptor Signaling and Trafficking by Arrestin-Src Kinase Interaction

Brant M. Wagener, Nicole A. Marjon and Eric R. Prossnitz

S3 Fig. Line scans of ligand, arrestin and either Rab11, AP-2 or AP-1 demonstrating colocalization in 633-6pep-stimulated Arr-2$^{-/-}$/-3$^{-/-}$ FPR cells. Arr-2$^{-/-}$/-3$^{-/-}$ FPR cells were transiently co-transfected with either Rab11-GFP ***(A)***, AP-2-GFP ***(B)*** or AP-1-GFP ***(C)*** and either empty mRFP vector (mRFP only), wild type arrestin-2-RFP (Arrestin-WT) or arr2-P91G/P121E-RFP (Arrestin-P91G/P121E). Cells were stimulated with 10 nM 633-6pep for 60 min and viewed by confocal fluorescence microscopy, followed by line intensity scanning in Zen software. Images are representative of three independent experiments.

S3A Fig

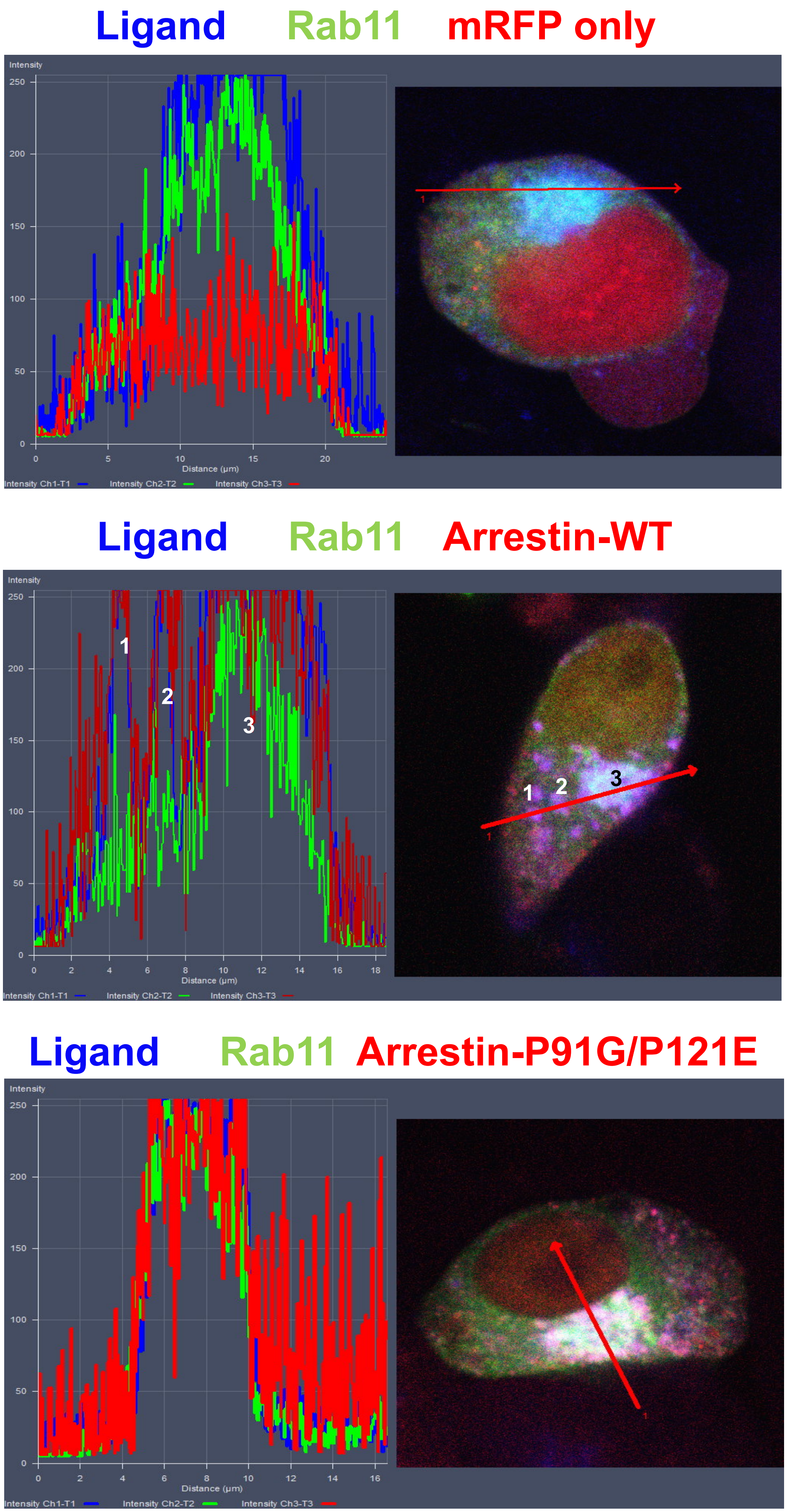

Ligand Rab11 mRFP only
Intensity
Distance (µm)
Intensity Ch1-T1
Intensity Ch2-T2
Intensity Ch3-T3
Ligand Rab11 Arrestin-WT
1
2
3
Ligand Rab11 Arrestin-P91G/P121E

S3B Fig

Ligand AP2 mRFP only

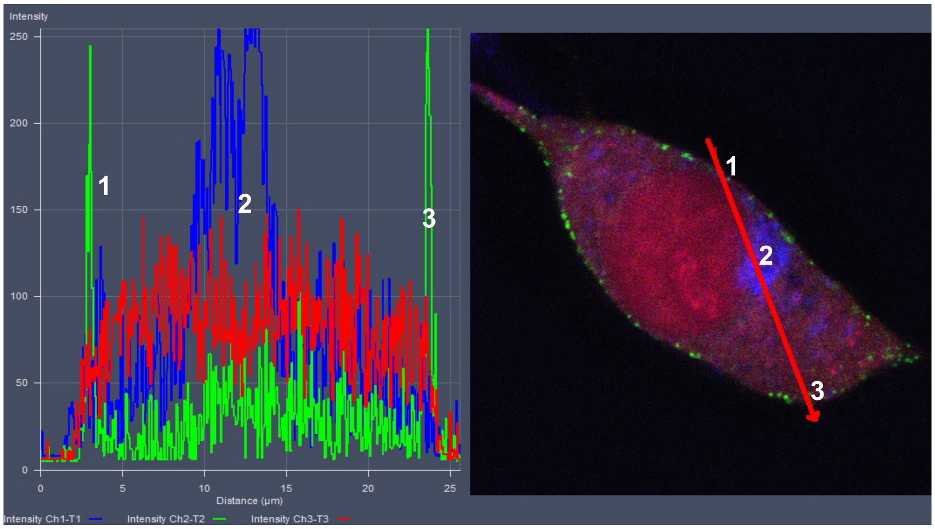

Ligand AP2 Arrestin-WT

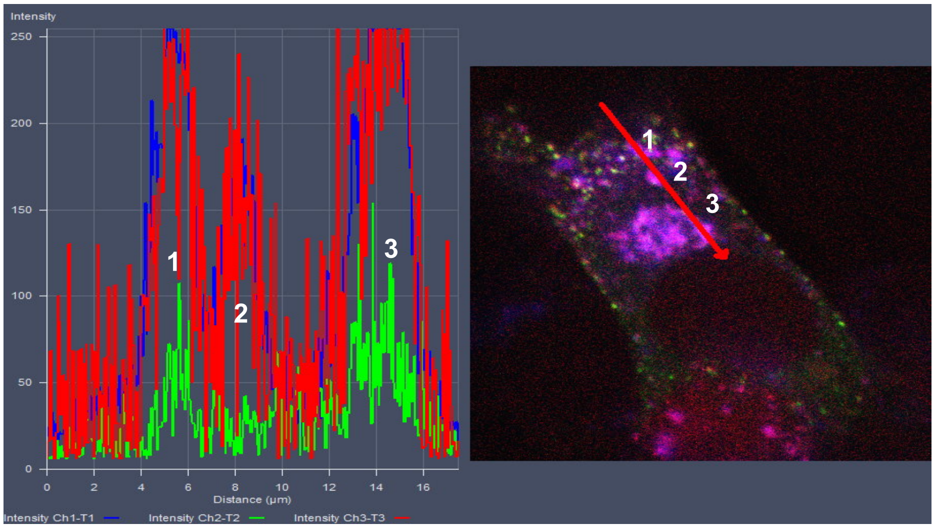

Ligand AP2 Arrestin-P91G/P121E

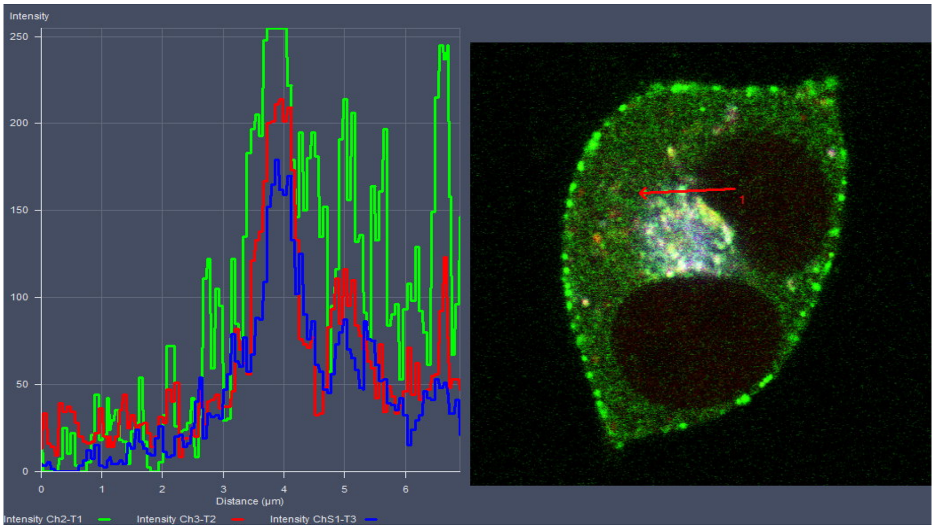

S3C Fig

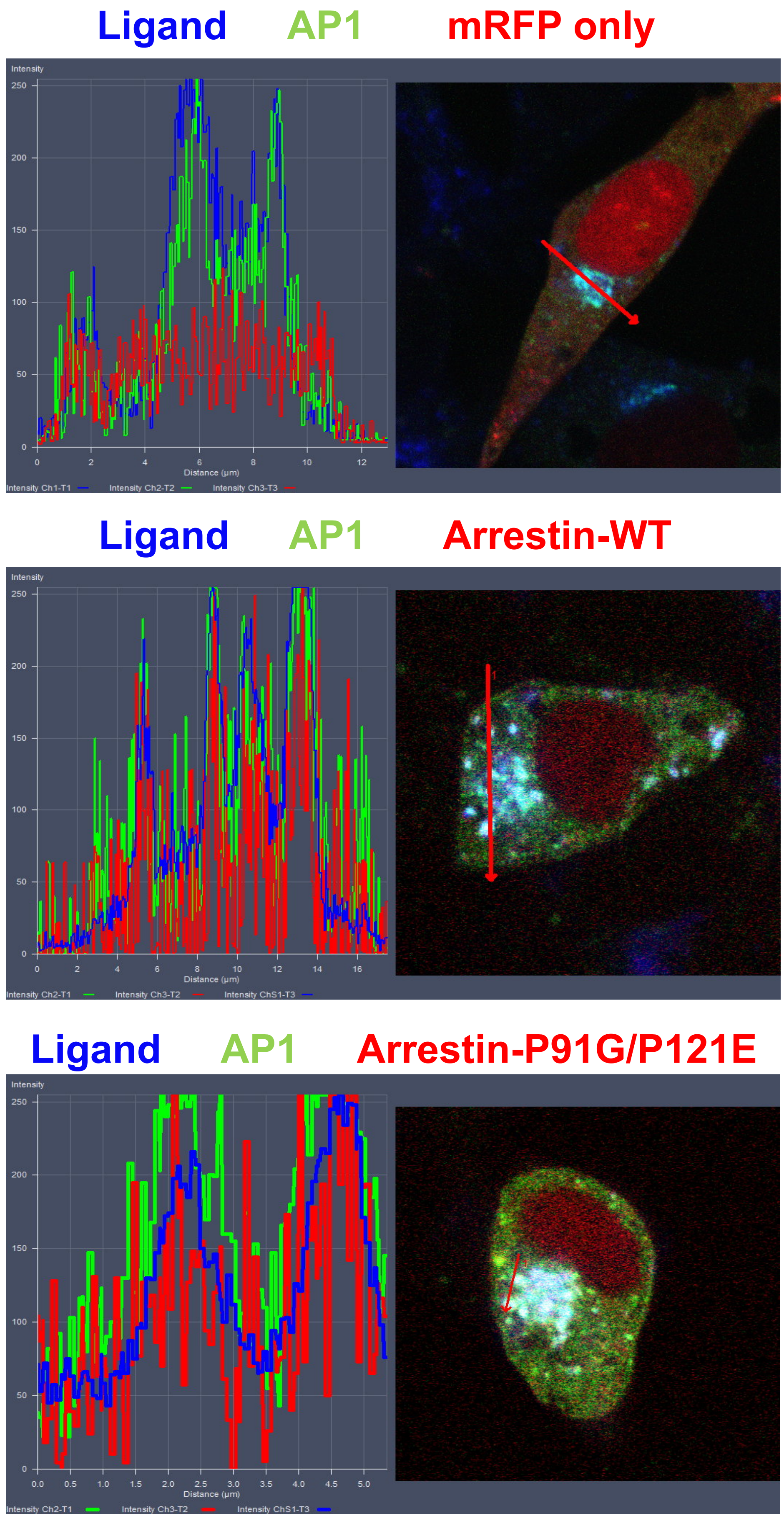
Ligand
AP1
mRFP only
Intensity
Distance (µm)
Intensity Ch1-T1
Intensity Ch2-T2
Intensity Ch3-T3
Ligand
AP1
Arrestin-WT
Intensity
Distance (µm)
Intensity Ch2-T1
Intensity Ch3-T2
Intensity ChS1-T3
Ligand
AP1
Arrestin-P91G/P121E
Intensity
Distance (µm)
Intensity Ch2-T1
Intensity Ch3-T2
Intensity ChS1-T3
